# pLM‐Repeat: Exploiting the sequence representations of protein language models for sensitive repeat detection

**DOI:** 10.1002/pro.70541

**Published:** 2026-04-07

**Authors:** Kaiyu Qiu, Andrei N. Lupas, Stanislaw Dunin‐Horkawicz

**Affiliations:** ^1^ Department of Protein Evolution Max Planck Institute for Biology Tübingen Tübingen Germany; ^2^ Institute of Evolutionary Biology, Faculty of Biology, Biological and Chemical Research Centre University of Warsaw Warsaw Poland

**Keywords:** AlphaFold database, bioinformatic tool, protein language model, repeat protein, sequence analysis

## Abstract

Duplication is an essential mechanism of molecular evolution, which operates across biological scales, from whole genomes to single basepairs. Its study is central to understanding protein evolution, but the detection of duplication events often becomes challenging over evolutionary time, due to the accumulating sequence divergence. The most sensitive sequence‐based protein repeat detection method, HHrepID, relies on the construction of multiple sequence alignments (MSAs) to enhance statistical signals of internal similarity and thus facilitate the detection of ancient duplications. However, such an alignment‐based approach comes at the expense of speed, severely limiting its applicability to large‐scale scans. Recent advances in protein representation learning have introduced sequence embeddings extracted from protein language models (pLMs) as a powerful and faster alternative to MSAs. Such representations have been shown to be effective in detecting distant sequence similarity, as exemplified by the pLM‐BLAST software developed in our group. In this study, we describe pLM‐Repeat, a pipeline built on top of pLM‐BLAST to identify repeat patterns encoded in sequence representations. pLM‐Repeat achieves comparable sensitivity to HHrepID in detecting the presence of repeats, while identifying many more repeat units and providing shorter runtimes, allowing us to detect novel repeat proteins in the AlphaFold Protein Structure Database with the aid of a pre‐filtering model trained on repeat protein representations. pLM‐Repeat is available as an open‐source tool at https://github.com/KYQiu21/plmrepeat.

## 1 INTRODUCTION

The first discovery of duplicated sequence patterns in proteins dates back to 1966 when Dayhoff and Eck identified a repeat pattern in ferredoxin and surmised that the protein had evolved by successive amplifications of the tetrapeptide A‐D/P‐S‐G (Eck and Dayhoff 1966). Over time, proteins with repetitive amino acid patterns have been found to be prevalent in all domains of life (Marcotte et al. 1999); indeed, a recent survey estimated that more than half of all proteins in the UniProt Knowledgebase (UniProtKB) contain at least one tandem sequence repeat (Delucchi et al. 2020). Repetitive sequences fold into structures that are typically stabilized by interactions between individual repeat units (RUs) and exhibit a wide range of structural diversity, from crystalline aggregates, through fibrous, solenoid or toroid structures, to beads‐on‐a‐string architectures (Kajava 2012). These unique structural features make repeat proteins a versatile platform for a wide range of studies, including protein folding (Petersen and Barrick 2021), disease pathogenesis (Deryusheva et al. 2023), molecular evolution (Chaudhuri et al. 2008), and macromolecular design (Parmeggiani and Huang 2017).

Given the importance of repeat proteins, their accurate detection remains a crucial bioinformatics task and is typically approached by methods based on the detection of sequence or structural similarity between RUs in a given protein (Pellegrini 2015). Currently, sequence‐based algorithms can be classified into several types. Methods based on short strings are adept at detecting short and highly repetitive tandem repeats, including XSTREAM using seed extension (Newman and Cooper 2007) and T‐REKS using k‐means clustering (Jorda and Kajava 2009). Fourier transform analysis is another method for detecting periodicity within sequences, but struggles with short repeats (Gruber et al. 2005). Alternatively, approaches such as TPRpred use prior knowledge by searching query sequences against pre‐computed profiles for repeat detection (Karpenahalli et al. 2007). However, the most widely used approach for identifying *de novo* repeats, including imperfect and long repeats, is self‐sequence alignment (SSA), that is, aligning the protein sequence of interest to itself using the Smith‐Waterman algorithm and inferring internal repeats from the resulting suboptimal local alignments. SSA‐based software such as RADAR typically takes a single sequence as input (Heger and Holm 2000). In contrast, HHrepID first searches a database for homologs and builds a multiple sequence alignment (MSA), from which suboptimal alignments are inferred by profile‐profile self‐comparison (Biegert and Söding 2008). The use of MSAs makes HHrepID a highly sensitive sequence‐based method, but reduces its speed and applicability to large scans. Meanwhile, structure‐based repeat detection adopts principles similar to sequence‐based methods but operates on protein structures instead of sequences. For instance, CE‐symm and SymD identify structural repeats by self‐alignment and circularly permuted structural alignment (Bliven et al. 2019; Kim et al. 2010), respectively. More recently, STRPsearch leverages the fast structural search engine Foldseek together with carefully curated libraries of representative repeat units, achieving high performance for knowledge‐based structural tandem repeat identification and expanding the coverage of RepeatsDB (Mozaffari et al. 2024).

In general, structure‐based approaches tend to outperform those based on sequences, because structures evolve more slowly, whereas internal repeat sequences often diverge rapidly and become difficult to detect (Schaper et al. 2012). However, it is important to note that significant sequence similarity typically indicates common ancestry, that is, homology, whereas structural similarity, especially when limited to smaller regions such as RUs, may be the result of convergent evolution, that is, analogy. With this in mind, we focused our work on sequence‐based repeat detection, which, although generally less effective, is more informative for studies aimed at understanding protein repeats from the evolutionary perspective (Alvarez‐Carreño et al. 2020).

In the search for faster ways to detect protein sequence repeats, we focused on the possibilities offered by protein language models (pLMs). These models, inspired by natural language processing techniques, are specialized deep learning models that are trained in a self‐supervised manner to understand the grammar of protein sequences. In recent years, numerical representations extracted from pLMs, such as ProtT5 (Elnaggar et al. 2021) and Evolutionary Scale Model (ESM) (Rives et al. 2021), have transformed various downstream applications in protein science, such as antibody design (Hie et al. 2024), prediction of protein‐protein interactions (Sledzieski et al. 2023), detection of transmembrane regions (Bernhofer and Rost 2022), and prediction of signal peptides (Teufel et al. 2022). Protein representations also contribute to applications involving sequence searching and processing, including MSA construction (McWhite et al. 2023), prediction of structural similarity from sequences (Hamamsy et al. 2023), and remote homology detection (Hong et al. 2024; Johnson et al. 2024; Kaminski et al. 2023; Pantolini et al. 2024). For example, tools like our own pLM‐BLAST utilize local similarity between sequence representations to identify regions indicative of potential homology, showcasing the effectiveness of these representations in uncovering evolutionary relationships. While pLM‐BLAST offers good speed due to its independence from the extensive sequence database searches required for generating MSAs, it shows performance comparable to HHpred (Söding et al. 2005), a highly sensitive method based on MSAs, and even detects statistical signals indicative of remote homology that HHpred may not capture.

The success of pLM‐BLAST and similar homology detection methods made us wonder if protein representations could also be used for repeat identification. In this study, we describe a new repeat detection method, called pLM‐Repeat, which replaces the time‐consuming MSA‐based self‐sequence alignment with the comparison of sequence representations using pLM‐BLAST. In our benchmarks, pLM‐Repeat shows promising performance compared to the most sensitive sequence‐based method, HHrepID, while providing faster analysis in most cases. To improve the applicability of pLM‐Repeat in large scans, we have trained an auxiliary neural network to rapidly detect potentially repetitive regions with patterns similar to those seen in known repeat protein domains deposited in the RepeatsDB. As an application of the pipeline, we scanned 682,563 sequences from the AlphaFold Database (AFDB) (Varadi et al. 2022) and identified 4,525 domains exhibiting potentially novel repetitive folding patterns.

## 2 MATERIALS AND METHODS

### 2.1 Datasets

The RepeatsDB database (version 3.2, https://repeatsdb.bio.unipd.it/) was used to generate a set of repeat proteins filtered to a maximum of 90% sequence identity and 80% coverage using MMseqs2 (version 13.4511, https://github.com/soedinglab/MMseqs2) (Paladin et al. 2021; Steinegger and Söding 2017), resulting in a dataset of 2056 repeat proteins. Both sequences and structures of the PDB entries of this dataset were retrieved using scripts provided on the PDB website (https://www.rcsb.org/downloads/). To construct a dataset of proteins without repeats, we adopted the protocol described by Alvarez‐Carreño et al. (Alvarez‐Carreño et al. 2020). First, we clustered PDB chain sequences at identity and coverage cutoffs of 30% and 80%, respectively, using MMseqs2. Then, entries marked as repetitive were excluded based on annotations from the InterPro database (Paysan‐Lafosse et al. 2023). Finally, the remaining structures were evaluated for the presence of internal symmetry using the SymD software (version 1.61, https://ccrod.cancer.gov/confluence/display/CCRLEE/SymD) (Kim et al. 2010). Proteins with a Z‐score of 4 or less were considered to be non‐symmetric and thus retained, resulting in a dataset of 8710 non‐repetitive proteins. From this set, 2100 proteins were randomly selected to create a negative dataset of comparable size to the positive dataset. These proteins were analyzed using HHrepID at a range of P‐values, and those reported as repetitive were manually reviewed and removed if they showed repeat patterns at the sequence or structure level, resulting in a total of 1977 non‐repetitive proteins. This sampled non‐repetitive protein dataset was used for the benchmark of repeat detection methods, while the full non‐repetitive set served as the negative dataset in the DeepRepeat model training.

For additional evaluation, we assembled two supplementary datasets derived from structure‐based annotations. The first set of 2627 repeat proteins was obtained from the DbStRiPs database following MMseqs2 filtering at 90% sequence identity and 80% coverage (Chakrabarty and Parekh 2022). The second dataset, comprising 2611 AFDB‐based repeat‐domain entries from the RepeatsDB (version 4) update (Clementel et al. 2025), was curated using a 30% identity and 80% coverage filter.

### 2.2 Implementation of pLM‐Repeat

**FIGURE 1:**
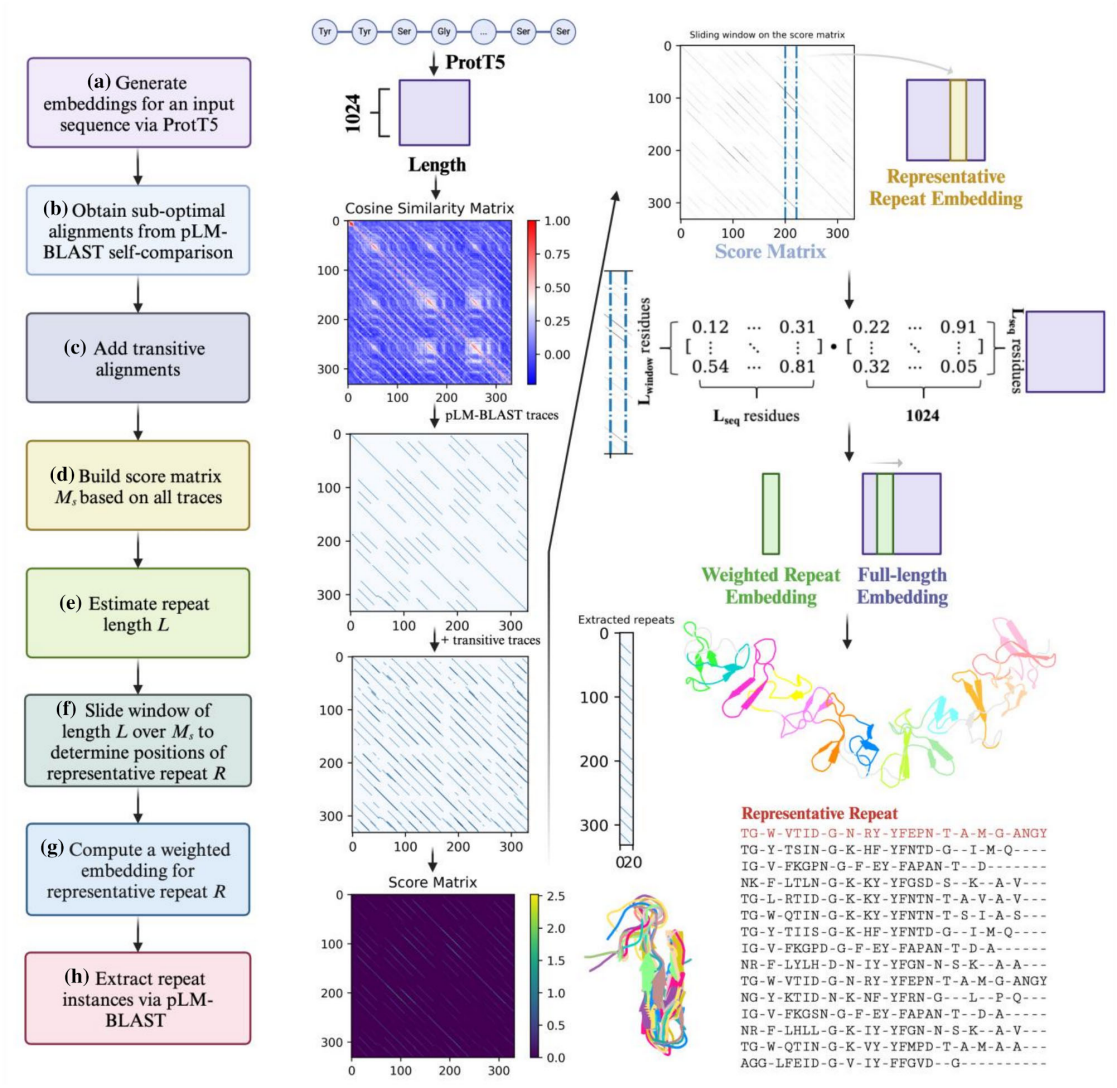
pLM‐Repeat pipeline. The main steps of pLM‐Repeat are shown as a flowchart, and the main intermediate outputs of the pLM‐Repeat workflow are presented using the domain 2QJ6_A as an example. The workflow of pLM‐Repeat includes (a) generating protein embeddings with the ProtT5 model, (b) retrieving suboptimal alignments by pLM‐BLAST self‐comparison, (c) enriching the alignment matrix by applying transitivity, (d) constructing the score matrix from collected traces, (e) estimating the repeat length, (f) determining the positions of the representative repeat, (g) computing a weighted repeat embedding, and (h) extracting repeat instances.

pLM‐Repeat was developed based on pLM‐BLAST, a tool for local homology detection based on direct comparison of sequence representations obtained from the ProtT5 protein language model (Kaminski et al. 2023). Unlike traditional sequence aligners such as BLAST, pLM‐BLAST replaces the fixed amino acid substitution matrix (e.g., BLOSUM62) with a context‐dependent similarity matrix derived from ProtT5 embeddings, which provides a similarity score for each individual residue pair. This context‐dependent substitution matrix is used to construct a score matrix, which is then subjected to a traceback procedure adapted from the Smith‐Waterman (SW) algorithm. Notably, pLM‐BLAST traces back from all positions in the score matrix, not just from the cell with the highest score as in the original SW procedure, to effectively report all significant traces. The pLM‐Repeat procedure involves the steps described below, with an example of domain 2QJ6_A showing intermediate outputs in Figure 1.

1. The input sequence of length *L*_seq_ is tokenized into individual amino acids, with each residue treated as a single token, resulting in a residue‐wise vector of shape (*L*_seq_, 1024) by the ProtT5 model (ProtT5‐XL‐UniRef50, https://huggingface.co/Rostlab/prot_t5_xl_uniref50) (Elnaggar et al. 2021). The generated raw embedding is then directly passed to pLM‐BLAST for self‐comparison in local mode with the following parameters: a window length of 15, a minimum span length of 15, a sigma factor of 2.0, a gap extension penalty of 0.0, and a self‐alignment score cut‐off of 0.3 (see Data S1, Supporting Information for the full parameter definitions and defaults). Sub‐optimal alignments that meet the defined length and score thresholds are collected, along with the corresponding pLM‐BLAST score for each alignment.
2. Transitivity has been shown to be a powerful approach in a number of sequence‐related bioinformatics algorithms, such as MSA construction and homology search (Söding et al. 2006). If residue *i* and residue *j* are aligned in one trace, and residue *j* and residue *k* are aligned in another trace, then residue *i* and residue *k* are assumed to be aligned in the third trace, called the transitive trace. For every two traces identified by pLM‐BLAST, all possible transitive traces are generated and scored according to the cosine similarity substitution matrix derived from the initial pLM‐BLAST self‐alignment. To speed up the procedure and avoid introducing too much noise, only one round of transitivity is applied to suboptimal alignments. The transitivity‐related thresholds and overlap settings are detailed in Data S1.
3. After obtaining all the traces, the score matrix *M*_*s*_ is constructed by calculating the score of each cell (*i*, *j*) based on the collected set of traces *T*,

$$M_{s}\left(i,j\right)=\sum_{\left(i,j\right)\in t,t\in T}\text{TraceScore}\left(t,i,j\right),$$

where *i* and *j* represent two residue positions, *t* denotes a trace containing the alignment of the *i*–*j* residue pair, and TraceScore represents the pLM‐BLAST score of that trace. The scores of all traces containing the *i*–*j* aligned pair are summed to determine the score of the corresponding cell (*i*, *j*) in the score matrix.

4 To estimate the repeat length, the scores of all cells located at the same distance (from 1 to *L*_seq_/2) to the diagonal of the score matrix are summed separately for each distance, and all distances with a score greater than 0 are considered as possible lengths to be evaluated in the following steps.
5 For each potential repeat length *l* stored in the previous step, a sliding window of *l* residues is used to scan along the scoring matrix *M*_*s*_. Following the procedure of HHrepID (Biegert and Söding 2008), we assume that conserved regions are more likely to be located in the middle of repeats, while the boundary regions are more prone to substitutions and indels. Considering this, we assign a weight to each column (residue) of a sliding window according to its position *p* to calculate the overall score,

$$w\left(p\right)=\left\{\begin{array}{c} p-\frac{1}{2},\;p\leq \frac{l}{2}\;\\ l-p+\frac{1}{2},\;\frac{l}{2}<p\leq l \end{array}\right.,$$

and for each possible repeat length *l*, the position *P*_*r*_ of the representative repeat is determined by maximizing the total score in the region covered by a sliding window,

$$\text{Score}\left(\text{window}\right)=\sum_{\left(i,j\right)\in \text{window}}w\left(j\right)M_{s}\left(i,j\right).$$

6 Once the position of the representative repeat is determined for a given repeat of length *l*, its embedding is calculated by weighting the original repeat embedding with the score matrix *M*_*s*_,

$$E_{\text{repeat}}=M_{\mathrm{sw}}E_{\mathrm{fl}},$$

where *M*_*sw*_ and *E*_*fl*_ represent the columns of the score matrix *M*_*s*_ corresponding to the residue range of the representative repeat and full‐length sequence embeddings, respectively. This scheme weights the embeddings of the corresponding sites using the columns of the score matrix, similar to creating a profile based on an MSA. In effect, the resulting weighted residue embeddings incorporate information collected in suboptimal alignments, thus improving the performance of the subsequent iterative repeat extraction step.

7 After obtaining the weighted embedding of the representative repeat, another local pLM‐BLAST comparison is performed to search it against the full‐length sequence embedding to extract repeat instances. Each identified repeat is then compared to the representative repeat embedding using pLM‐BLAST in global mode to derive the pairwise alignment and its score. These pairwise alignments are then combined to produce a multiple alignment. Finally, the results obtained for different estimated repeat lengths *l* are compared based on selected evaluation metrics and the one with the best metrics is reported (see Data S1 for the default metric and available options). Throughout this study, the total number of correctly predicted (i.e., part of the ground truth repeat) residues in all reported repeats was used as the benchmark metric, except for the analysis shown in Figure 4, where the repeat length was determined according to the average coverage of reported repeats against the representative repeat.
8 Upon completion of a search round, the score matrix *M*_*s*_ is updated by masking all residue positions involved in the repeat regions identified in that round. Steps 4–7 are then repeated with the updated score matrix to identify possible additional repeat regions. An example of the analysis of a protein containing more than one repetitive region is shown in Figure S1.

### 2.3 Benchmark of protein repeat detection software

In addition to pLM‐Repeat, two self‐alignment algorithms were selected for the performance benchmark: RADAR (version 1.3, https://github.com/AndreasHeger/radar) (Heger and Holm 2000), which operates on single sequences, and HHrepID (http://ftp.tuebingen.mpg.de/pub/protevo/HHrepID/) (Biegert and Söding 2008), which uses MSAs as input. Additionally, we evaluated the performance of HHrepID with a single sequence (no MSA) as input. Domains from both the positive and negative datasets (see section 2.1) were analyzed in each software. The benchmark was performed at two levels: protein level, and repeat level. In the protein‐level benchmark, a protein was identified as repeat‐containing by a given method if more than half of its predicted repeats correctly aligned with at least one another predicted repeat from the same method. At the repeat level, each predicted repeat was considered correct if it correctly aligned with at least one ground truth repeat annotated in the RepeatsDB database. For non‐repetitive proteins identified as repeat proteins, we took the number of repeats detected as the number of false positive repeats. In both benchmark modes, a correct alignment was defined as a structural alignment with a length‐normalized TM‐score greater than 0.5 and a sequence coverage greater than 50% provided by TM‐Align (Zhang and Skolnick 2005). Repeat structures corresponding to the detected residue ranges were extracted using the Biopython package (Cock et al. 2009), following the residue mapping dictionary provided in the localpdb package (Ludwiczak et al. 2022). Repeat sequence alignments were conducted in the pairwise module of Biopython.

**FIGURE 2:**
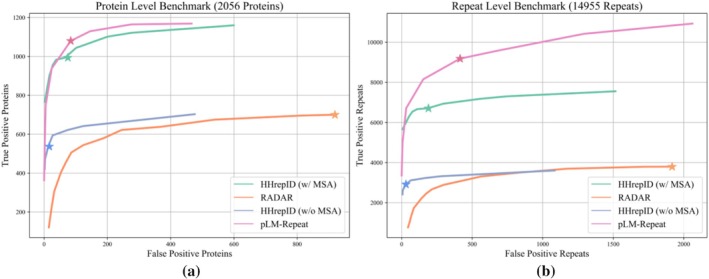
Benchmark results. Performance benchmark of sequence‐based repeat detection methods. Repeat family *p*‐value thresholds, repeat score thresholds, and self‐sequence alignment score thresholds were evaluated across a range of values for HHrepID (with or without MSA), RADAR, and pLM‐Repeat, respectively. The performance of each method at default settings is marked with an asterisk. Benchmarking was performed at the protein (a) and repeat (b) levels (see section 2 for details).

MSAs for all proteins in the repeat and non‐repeat datasets were generated by searching each sequence against the UniRef30 database (version 2023‐02, https://colabfold.mmseqs.com/) (UniProt Consortium 2023) using HHblits with default settings (Remmert et al. 2012). A series of repeat score thresholds, repeat family P‐value thresholds, and self‐sequence alignment score thresholds were evaluated for RADAR, HHrepID, and pLM‐Repeat, respectively, to derive the benchmark results shown in Figure 2. We also tested CE‐symm (version 2.2.2, https://github.com/rcsb/symmetry/tree/master) (Bliven et al. 2019), a state‐of‐the‐art structure‐based repeat detection software, on the same datasets using default settings. To compare CE‐symm with pLM‐Repeat equipped with structure embeddings, the structures of the proteins included in the benchmark dataset were converted into embeddings by two inverse folding models, ESM‐IF (Hsu et al. 2022) and MIF (Yang et al. 2023), respectively. These residue‐wise embeddings were used as inputs to pLM‐Repeat with the transitivity turned off to avoid introducing noise and other settings left as defaults.

We performed the speed comparison by analyzing proteins of different lengths using RADAR, HHrepID, and pLM‐Repeat. The speed test was performed on the same system equipped with an AMD EPYC 7742 64‐core CPU. Runtime for each query in each software was averaged in three replicates.

### 2.4 Implementation of DeepRepeat

To enable fast pre‐filtering in large scans, we trained a neural network called DeepRepeat to identify repeats with patterns similar to those found in known repeat proteins (Figure 5a; additional implementation and training details are provided in Data S1). DeepRepeat uses slightly modified light attention architecture proposed by Stärk et al. (Stärk et al. 2021). The model takes as input a per‐residue embedding, which is also the input of pLM‐BLAST. The embedding is transformed by two separate 1D convolution layers, both with a filter size of 9 and an output channel of 1024. The output of the first convolution layer is followed by a Softmax layer to generate attention distributions, while the output of the second convolution layer is followed by a Dropout layer and results in feature maps. The Hadamard product of the attention distributions and feature maps is summed along the sequence length dimension to generate the weighted sum, which is then concatenated with the feature maps after the MaxPool operation. The resulting fixed shape embeddings of the input samples are then fed into a linear layer for binary classification. The model was trained using the Adam optimizer at a learning rate of 1 × 10^−6^, and the dataset was split into training and test sets at a ratio of 9:1. MMseqs2 clustering was applied with an identity and coverage threshold of 50% and 80%, respectively, to filter out highly similar sequences from the test set that were present in the training set. Early stopping was implemented to halt training when the test loss ceased to decrease for 20 epochs. Binary cross‐entropy loss was used for training. Precision, recall, and F1 score were evaluated to assess the performance of the model,

$$\text{Precision}=\frac{\mathrm{TP}}{\mathrm{TP}+\mathrm{FP}},$$

$$\text{Recall}=\frac{\mathrm{TP}}{\mathrm{TP}+\mathrm{FN}},$$

$$F1-\text{score}=2\times \frac{\text{Precision}\times \text{Recall}}{\text{Precision}+\text{Recall}}.$$

To explore the potential interpretability of the neural network, raw attention weights with a shape (*L*_seq_, 1024) were extracted for each protein of length *L*_seq_ before applying the Softmax layer. The average across the 1024 dimensions was assigned as the attention score for each residue and mapped to protein structures for visualization. We also examined the embeddings of all repeat and non‐repeat proteins obtained from the Concat layer, which is the last layer of the light attention module. Each embedding from the Concat layer has a fixed size of 2048 regardless of sequence length. All protein embeddings were projected into 2D space and visualized by the Uniform Manifold Approximation and Projection (UMAP) framework using the UMAP package (McInnes et al. 2018). The model was implemented in the PyTorch framework (version 1.13.1).

### 2.5 Scan on the dark entries in the AFDB90v4 database

The AFDB90v4 database was downloaded from the Uniprot3D website (https://uniprot3d.org/) (Durairaj et al. 2023). Each protein in this database is assigned a “functional brightness,” defined as the coverage of the sequence with annotations of homologs, ranging from 0 to 1. 682,563 sequences with low functional brightness (≤0.1) were collected from the AFDB90v4 database, converted to embeddings using the ProtT5 model, and passed to the DeepRepeat model. Entries recognized as positive by the model (using a threshold of 0.5) were further clustered to 50% sequence identity and 80% coverage using MMseqs2. We assessed their sequence novelty by searching them against the ECOD70 database (version 20230309, http://prodata.swmed.edu/ecod/index_pdb.php) (Cheng et al. 2014) using HHSearch in the single‐sequence searching mode (version 3.3.0, https://github.com/soedinglab/hh-suite) (Steinegger et al. 2019) with default settings and discarding those reporting hits with a probability greater than 30%. The predicted structures of the remaining proteins were retrieved using the AlphaFold database API and searched against the PDB100 database (https://foldseek.steineggerlab.workers.dev/) using Foldseek (version 1.3c64211, https://github.com/steineggerlab/foldseek) (Van Kempen et al. 2024) with an E‐value threshold of 0.1 in the 3Di/AA alignment mode to identify structures that had no similar counterparts in the PDB. Given our interest in globular repeat folds in this study and the prevalence of coiled structures observed in the dataset, we further applied DeepCoil2 (version 2.0.2, https://github.com/labstructbioinf/DeepCoil/tree/master) (Ludwiczak et al. 2019), a deep learning‐based coiled‐coil predictor, with a stringent filtering criterion, excluding entries where the coiled‐coil residue fraction exceeded 30%. These filters resulted in a data set of 4,525 proteins of potential interest for further manual inspection and analysis (Data S2).

### 2.6 Analysis of selected repetitive folds

We examined several selected proteins from the filtered set generated above using a variety of tools: homology search using HHpred and pLM‐BLAST, repeat detection using HHrepID and pLM‐Repeat, structure search using Foldseek, and sequence clustering using MMseqs2. The majority of these analyses, with the exception of our newly developed tools and Foldseek, were performed using the MPI bioinformatics toolkit (https://toolkit.tuebingen.mpg.de/) (Zimmermann et al. 2018).

### 2.7 Visualization

Structures were visualized with PyMOL 3.0. Figures 1 and 5a were generated using BioRender.com. Other figures were plotted using the Matplotlib and seaborn packages.

## 3 RESULTS

### 3.1 pLM‐Repeat benchmark

pLM‐Repeat identifies repeat patterns in a given sequence based on local self‐alignments obtained with pLM‐BLAST (see section 2 for details). We evaluated the performance of pLM‐Repeat on the compiled set of 2056 repetitive and 1977 non‐repetitive protein sequences along with two other sequence‐based self‐alignment methods, HHrepID and RADAR. In contrast to RADAR, which works with a single sequence input, HHrepID can take either a sequence as input or an MSA derived from that sequence, the latter approach providing significantly better accuracy at the expense of the time required to construct an MSA. The benchmark procedure involves evaluating pLM‐Repeat, RADAR, and HHrepID (in MSA and no‐MSA modes) under two scenarios, first to assess the ability to discriminate between repetitive and non‐repetitive proteins and second to examine the accuracy in predicting individual repeat units. The key parameter of pLM‐Repeat is the alignment similarity threshold (0 to 1, default 0.3), which filters suboptimal alignments detected by pLM‐BLAST for further processing in the pLM‐Repeat pipeline (Figure 1).

In the first protein‐level benchmark, both pLM‐Repeat and standard HHrepID significantly outperformed the single sequence‐based methods RADAR and HHrepID without MSAs, identifying approximately twice as many repeat proteins with an average false positive rate of approximately 10% (Figure 2a). With default settings, pLM‐Repeat showed comparable performance to HHrepID, both identifying 1080 and 994 correct repeat domains, respectively, with a similar number of false positives of 84 and 66, respectively. Notably, HHrepID can still detect 766 repeat proteins without false positives at a very stringent *p*‐value threshold of 1 × e^−13^, while 363 repeat domains can be identified without false positives at a score threshold of 0.5 in pLM‐Repeat, indicating a better false positive control in HHrepID due to the statistical evaluation framework used.

All benchmark cases where at least one of the methods running in default mode made a correct prediction were collected and categorized based on the RepeatsDB protein fold classes (Table 1). We also included predictions from a structure‐based software, CE‐symm, to provide a reference point for assessing potential bias in the evaluated methods. Among all sequence‐based methods, HHrepID and pLM‐Repeat showed the best performance in most fold classes, the only exception being TIM‐barrels, for which the simplest of the methods, RADAR, provided the best results (Table 1). pLM‐Repeat showed a tendency to perform better in certain protein fold classes, such as β‐propellers, where it correctly identified 259 (70.0%) domains, approaching the performance of the structure‐based method CE‐symm. Interestingly, pLM‐Repeat also performed best in two repeat protein folds with highly diverse repeat sequences but obvious structural repetitive patterns, β‐barrels/hairpins and α‐solenoids, detecting 41 (17.9%) and 336 (73.5%) domains, respectively, compared to 18 (7.9%) and 269 (58.9%) by the second‐best method, HHrepID (see Figures S2 and S3 for example of pLM‐Repeat predictions). On the other hand, HHrepID demonstrated superior performance in identifying β‐solenoid, achieving 101 correct detections (56.1%), followed by pLM‐Repeat, which ranked second in sequence ‐based methods with 71 correct predictions (39.4%).

**TABLE 1:** Number of correctly detected proteins on RepeatsDB dataset based on fold classes.

Repeat protein fold	pLM‐Repeat	HHrepID	HHrepID‐single	RADAR	CE‐symm
β‐Solenoid (180)	71 (39.4%)	**101** (56.1%)	43 (23.9%)	62 (34.4%)	80 (44.4%)
α/β Solenoid (150)	104 (69.3%)	**112** (74.7%)	100 (66.7%)	60 (40.0%)	106 (70.1%)
α‐Solenoid (457)	**336** (73.5%)	269 (58.9%)	145 (31.7%)	217 (47.5%)	306 (67.0%)
β Hairpins (40)	17 (42.5%)	**25** (62.5%)	14 (35.0%)	13 (32.5%)	17 (42.5%)
Box (57)	40 (70.2%)	**44** (77.2%)	0 (0.0%)	14 (24.6%)	44 (77.2%)
TIM‐Barrel (370)	45 (12.2%)	28 (7.6%)	8 (2.1%)	**57** (15.4%)	101 (27.3%)
β‐barrel/hairpins (229)	**41** (17.9%)	18 (7.9%)	1 (0.4%)	9 (3.9%)	54 (23.6%)
β‐Propeller (370)	**259** (70.0%)	216 (58.4%)	126 (34.1%)	141 (38.1%)	261 (70.5%)
α‐Barrel (28)	**24** (85.7%)	21 (75.0%)	9 (32.1%)	0 (0.0%)	22 (78.6%)
α/β Trefoil (49)	38 (77.6%)	**39** (79.6%)	18 (36.7%)	22 (44.9%)	31 (63.3%)
Prism (40)	29 (72.5%)	**33 (82.5%)**	3 (7.5%)	20 (50.0%)	40 (100%)
Beads‐on‐a‐string (109)	71 (65.1%)	**79 (72.5%)**	66 (60.6%)	64 (58.7%)	40 (36.7%)

*Note*: All methods were run with default settings to obtain the statistics for each fold class (with the number of domains in parentheses) in this table. The Beads‐on‐a‐string category consolidates five related fold classes used in RepeatsDB: α‐Beads, β‐Beads, α/β‐Beads, β‐Sandwich‐Beads, and α/β‐Sandwich beads. The Prism category combines the α/β‐Prism and Aligned‐Prism folds. Statistics for the method that achieved the best performance among all sequence‐based approaches are highlighted in bold. CE‐symm performance is included for ease of comparison.

Given the poor performance of pLM‐Repeat on TIM‐barrels, we ran the benchmark with the pLM‐Repeat similarity threshold lowered to 0.25. This change increased the false positive rate from 4.2% to 13.9%, but also resulted in 85 more correctly predicted proteins, mostly TIM‐barrels, increasing from 45 (12.2%) to 107 (29.0%; see Table S1 for all fold classes). Visualization of the results obtained with the less stringent threshold shows that pLM‐Repeat successfully identified the βα repeats in most of the 62 additionally detected TIM barrels, despite their low sequence identity, as shown in Figure S4. The promising performance of pLM‐Repeat on these challenging targets underscores its potential to reveal the evolutionary basis of observed structural periodicity, even in cases where the RUs have diverged almost beyond recognition.

Figure 2b shows the results of the second benchmark, which focuses on the detection of individual repeats by comparing them to the reference RUs obtained from RepeatsDB. Similar to the first benchmark, both pLM‐Repeat and HHrepID report more than twice as many correct repeats as RADAR and HHrepID without MSAs. In addition, HHrepID again shows good sensitivity with a low false positive rate, achieving up to 6710 correct repeats versus only 190 incorrect repeats with default settings. In contrast, pLM‐Repeat run with a default threshold of 0.3 correctly detects a much larger number of repeats (9182), while reporting 414 false positives. This discrepancy may be due to the fact that pLM‐BLAST excels at detecting short but significant local alignments and therefore includes a wider range of repeats within domains.

In addition to the RepeatsDB benchmark based on manually curated repeat annotations, we evaluated all methods on two repeat datasets derived from automatic structure‐based annotations. The first dataset of 2627 repeat sequences was obtained from the DbStRiPs database (Chakrabarty and Parekh 2022), which was constructed using PRIGSA2, a graph‐based structural repeat detection algorithm. The performance of all methods at their default thresholds shows similar trends to the main benchmark; pLM‐Repeat and HHrepID (MSA mode) were on par with each other and outperformed the other methods (Table S2). pLM‐Repeat detects slightly more repeats than HHrepID (MSA mode), likely reflecting the composition of DbStRiPs, which contains a larger proportion of α‐solenoid repeats, for which pLM‐Repeat also performed better in the main benchmark. For example, pLM‐Repeat identifies 19.4 percentage points (pp) more HEAT repeats (α‐solenoid) and 15.3 pp more tetratricopeptide repeats (α‐solenoid); however, it detects 1.8 pp fewer leucine‐rich repeat domains (α/β‐solenoid) and 16.9 pp fewer left‐handed β helix domains (β‐solenoid) compared to HHrepID (MSA mode). The second dataset was prepared based on the recent update of RepeatsDB that expands coverage by incorporating over 30,000 repeat domains from AFDB identified with STRPsearch (Clementel et al. 2025; Mozaffari et al. 2024). For this dataset, we used a filtered set of 2611 entries and found that pLM‐Repeat again performs comparably to HHrepID (MSA mode), detecting 1721 (65.9%) and 1746 (66.9%) repeat domains, respectively (Table S3). It is also worth noting that despite comparable domain‐wise sensitivity of the two methods, pLM‐Repeat detects substantially more repeat units (63.5% vs. 47.5%). These results indicate that pLM‐Repeat matches HHrepID (MSA mode) also on repeat sets defined purely by automated, structure‐based annotations.

Finally, we compared the run times of the benchmarked methods using proteins of different lengths (Table 2). RADAR was the fastest, providing results within 0.1 s for all sequences, followed by HHrepID without MSA, which completed the calculations within seconds. In contrast, the standard HHrepID took minutes to complete each job, while pLM‐Repeat performed the corresponding analyses significantly faster. For example, pLM‐Repeat and HHrepID took 24.7 and 272.2 s, respectively, for the 2QJ6_A domain of 332 residues.

**TABLE 2:** Speed comparison of sequence‐based repeat detection methods.

Query (length)	pLM‐Repeat	HHrepID	HHrepID‐single	RADAR
6A57_A (140)	7.4 s	285.3 s	1.2 s	0.1 s
4DB6_A (211)	9.4 s	271.5 s	1.8 s	0.1 s
2QJ6_A (332)	24.7 s	272.2 s	2.4 s	0.1 s
5AMS_A (431)	23.1 s	606.7 s	0.6 s	0.1 s

*Note*: All software was used with default settings to obtain the statistics in this table. The runtimes of pLM‐Repeat and HHrepID include the step of obtaining protein sequence embeddings and multiple sequence alignments, respectively. HHrepID on a single sequence took only 0.6 s to complete the procedure on the 5AMS_A domain because it detected a few suboptimal alignments.

### 3.2 Examples of pLM‐Repeat predictions

**FIGURE 3:**
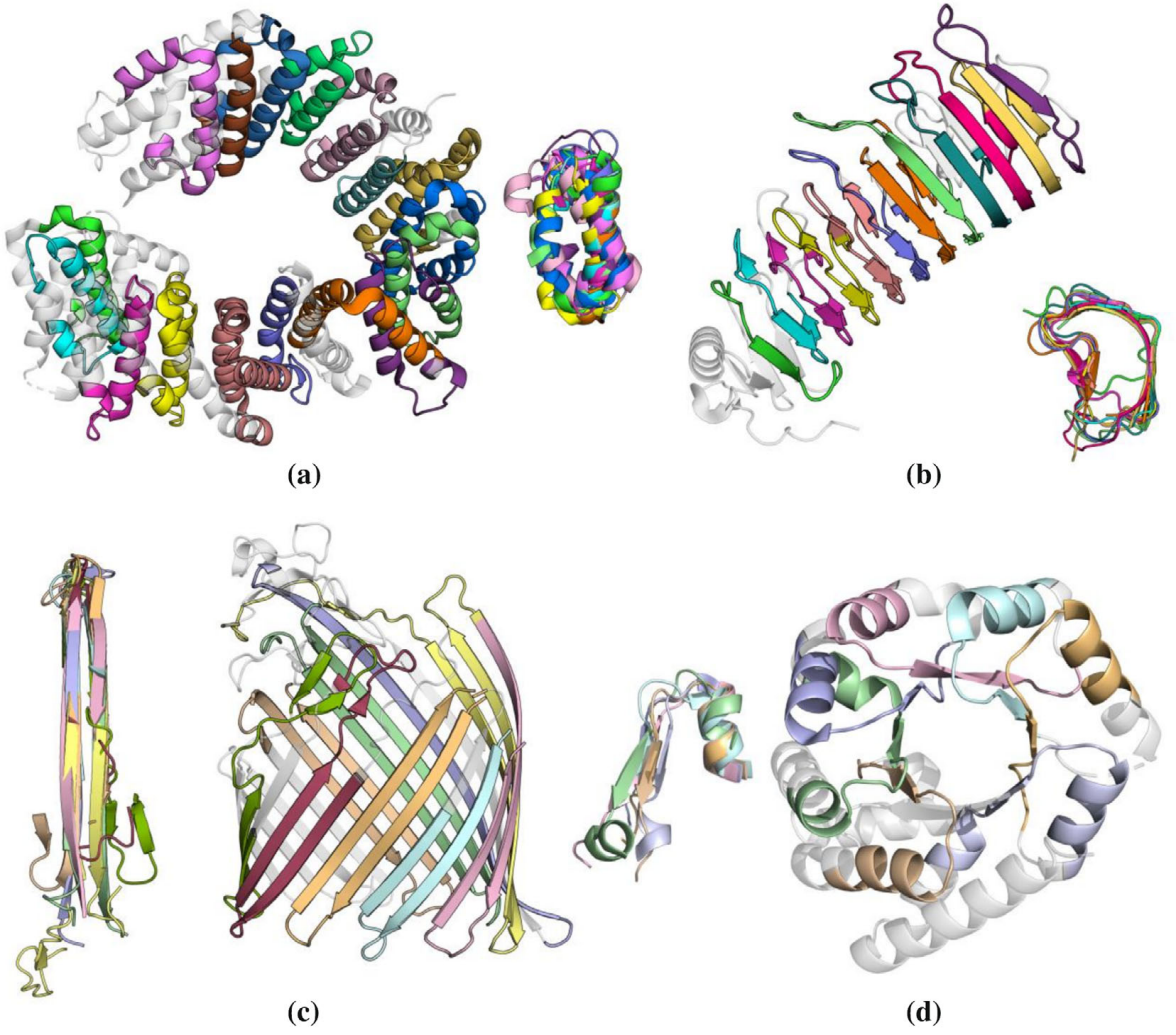
Performance cases of pLM‐Repeat. Repeat detection results for (a) an Armadillo α‐solenoid domain 2X19_B (repeat identity 19.9%), (b) a β‐solenoid domain 7C7D_A (20.5%), (c) an outer membrane β‐barrel 5NEC_A (18.9%), and (d) a TIM barrel 3NAV_A (19.2%). Structures are colored according to the identified repeat regions, with repeats superposed to show structural similarity. Residues not included in the detected regions are shown in white with semi‐transparency. The detailed analysis results of these examples can be found in Data S1.

In this section, we demonstrate the performance of pLM‐Repeat in detecting repeat units with low pairwise sequence identity in proteins of different folds. Key intermediate outputs through the analysis of these domains can be found in Data S1. For example, chain B of protein PDB:2X19 is an Armadillo repeat consisting of 22 repeats with an average sequence identity of 19.9% (Figure 3a). pLM‐Repeat successfully detected 17 repeats, most of which were α‐hairpins, while HHrepID with 3 rounds of HHblits search to generate MSAs failed to report any repeats. We also applied pLM‐Repeat to a pectin lyase‐like β‐solenoid domain (PDB: 7C7D) (Figure 3b). Despite the low average internal sequence identity of 20.5%, 12 out of 13 repeats were correctly recognized, while HHrepID identified 10 repeats (Figure S5a). In a 22‐stranded β‐barrel with an average repeat identity of 18.9%, pLM‐Repeat reported 9 repeat fragments, of which 8 corresponded to superimposable β‐hairpins (Figure 3c). In the same sequence, HHrepID recognized 6 repeat instances after constructing an MSA with 4 rounds of HHblits (Figure S5b). Finally, analysis of a tryptophan synthase TIM barrel revealed 7 β‐α fragments with an average sequence identity of 19.2% (Figure 3d), of which no repeats were identified by HHrepID.

**FIGURE 4:**
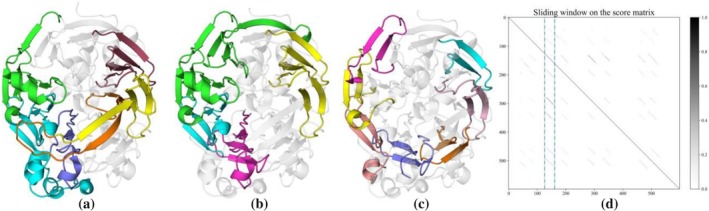
Performance comparison. Performance comparison between RADAR (a), HHrepID (b), and pLM‐Repeat (c) on an 8‐bladed propeller domain (PDB: 1W6S). Structures are colored according to the identified repeat regions, while residues not included in the detected regions are shown in white with semi‐transparency. (d) Score matrix with the determined sliding window. Eight traces are clearly visible within the region of the sliding window (see Data S1 for detailed outputs of pLM‐Repeat on this domain).

The example of the 8‐blade propeller domain (PDB:1W6S) with highly divergent repeats (20.8% average sequence identity and a significant number of insertions) demonstrates the potential of the pLM‐Repeat in tackling hard targets. RADAR struggled with this domain due to the interference from massive indels, resulting in structurally dissimilar and inaccurate repeat sequences (Figure 4a). On the other hand, HHrepID (MSA mode) performed better, identifying four repeats (Figure 4b); however, only two of these corresponded to a 4‐stranded β‐meander propeller blade, while the rest encompassed more than one RU (shown in green and yellow). In contrast to the above methods, pLM‐Repeat successfully detected seven out of eight RUs (Figure 4c,d); however, it missed complete blades and instead detected only the β3–β4 hairpin of each β‐meander. This limitation is due to insertions in the loop between β2 and β3 in certain blades. Nevertheless, in this case, only pLM‐Repeat provided a result in which the predicted repeat units, even though incomplete, are structurally consistent. The robustness of pLM‐Repeat shown in these challenging targets is further supported by the analysis of embedding self‐alignment scores. As expected, across our curated RepeatsDB dataset, the self‐alignment scores from pLM‐BLAST correlate with pairwise repeat sequence identity and 76.5% cases with <20% sequence identity still exhibit self‐alignment scores above 0.3, indicating that our method can capture similarities that go beyond primary‐sequence conservation (Figure S6a). This is further emphasized by the observation that diverged repeats with <20% sequence identity show markedly higher scores than non‐repeat proteins (Figure S6b), often exceeding 0.3, the default pLM‐Repeat score threshold.

### 3.3 Distinguish between proteins with and without repeats using a deep learning model

**FIGURE 5:**
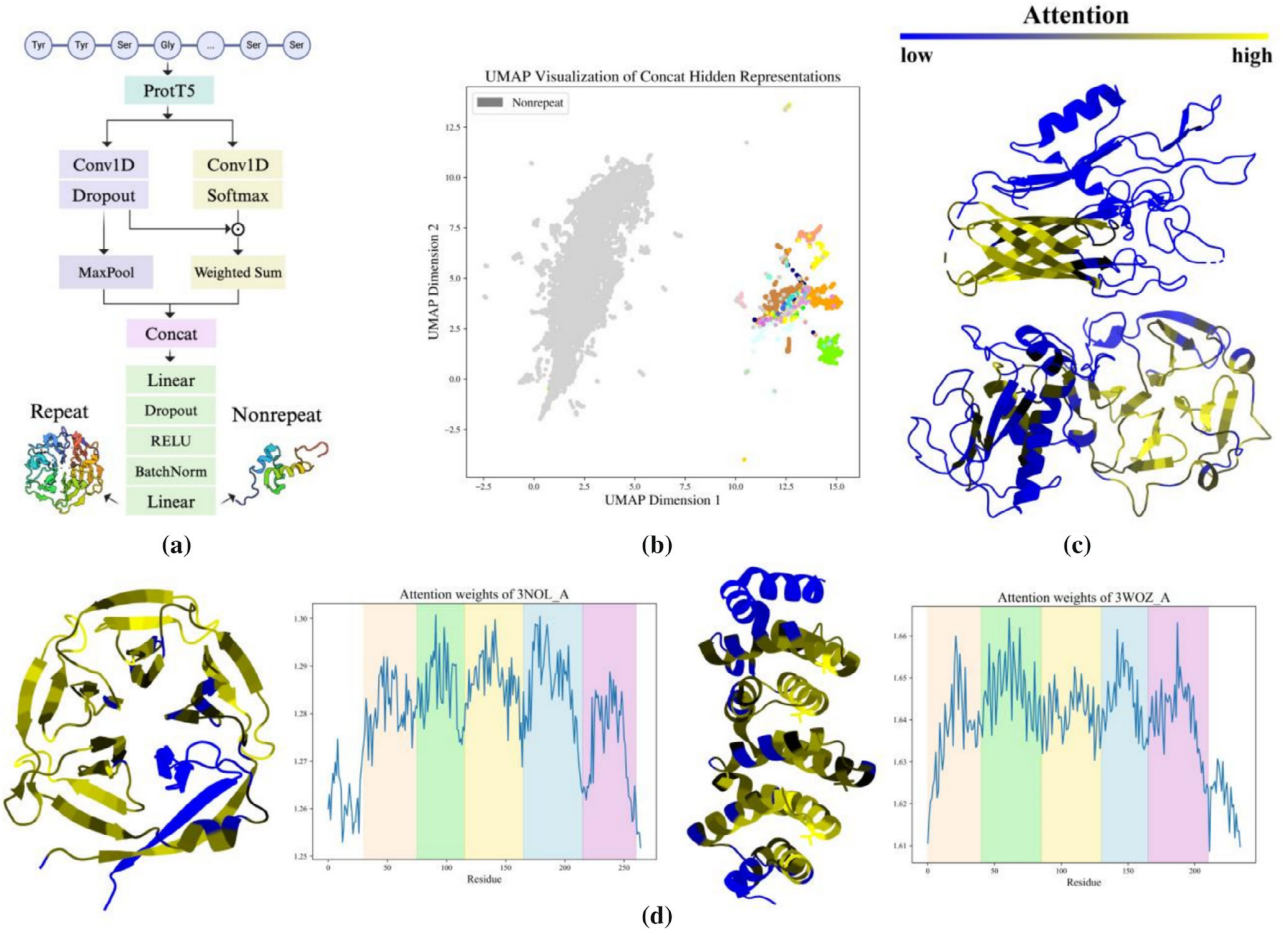
A neural network for distinguishing between known repetitive and non‐repetitive proteins. (a) The model architecture, including a light attention module and a prediction layer. (b) The UMAP visualization of all proteins with and without repeats in the compiled dataset. Nodes of non‐repeat proteins are colored in gray, while nodes of repeat proteins are colored in different colors based on fold class. (c) Protein structures colored based on attention scores, with blue and yellow representing low and high attention scores, respectively. Repeat domains in 3UAQ_A (top) and 1FBL_A (bottom) show higher attention scores than their non‐repeat counterparts. (d) Repeat proteins 3NOL A (left) and 3WOZ A (right) show periodic attention patterns, with x‐axis and y‐axis denoting the residue index and attention scores, respectively. Vertical shaded regions correspond to individual repeating units in the protein sequence.

While speed improvements have been achieved with pLM‐Repeat, it is not efficient enough for large‐scale scans of sequence databases such as UniProt. To overcome this limitation, an auxiliary pre‐filtering model, DeepRepeat, was developed to speed up such scans by limiting the number of sequences fed to pLM‐Repeat (Figure 5; see section 2 for details). The model is a neural network trained using curated repeat proteins from the RepeatsDB database together with the compiled negative dataset to detect whether input sequences exhibit patterns resembling those of known repeat proteins. Conceptually, our model follows the same knowledge‐based rationale as tools like the profile‐based TPRpred (Karpenahalli et al. 2007). To evaluate the classification potential of DeepRepeat, its internal representations were analyzed. These internal representations provide a numerical description of the input sequences in the context of the knowledge captured by the model. Visualization of the DeepRepeat Concat layer representations obtained for the training sequences (Figure 5b) shows a clear separation between repeat and non‐repeat proteins, which is essential for accurate classification. Indeed, the model achieved an F‐score of 0.902 on the test set (with a precision of 0.851 and a recall of 0.961), demonstrating its effectiveness in detecting embedding patterns similar to known repeat proteins used for training. Finally, since the model incorporates a light attention module (Stärk et al. 2021), it is possible to extract its attention scores and indicate which regions of a given protein were essential for the prediction. For proteins containing both repetitive and non‐repetitive regions, the former had significantly higher attention scores, although proteins involved in the training dataset were not explicitly split into individual domains, suggesting the potential of the model not only to provide a binary classification, but also to capture the regions containing repeats. Interestingly, we noticed that the attention weights for some repeat regions showed a clear periodicity pattern (Figure 5d), further indicating that the repetitive patterns were successfully recognized by the model.

### 3.4 Gallery of AFDB90v4 repeat proteins

To demonstrate the practical application of the DeepRepeat model coupled with the pLM‐Repeat method, we performed a large‐scale scan of the AFDB90v4 database (Durairaj et al. 2023). The AFDB90v4 database contains high quality AlphaFold2 models (pLDDT>90) for UniRef database sequences that share no more than 50% identity. In addition, each sequence within AFDB90v4 is assigned a “functional brightness” index, ranging from 0 to 1 according to the annotation coverage provided in different databases. We focused on 682,563 entries with functional brightness scores lower than 0.1, as these functionally unknown proteins are more likely to represent undefined families. The ProtT5 embeddings calculated for these proteins were first fed into the DeepRepeat model, and then 73,324 entries predicted as positive were subjected to further clustering and analysis to evaluate repeat patterns and assess both sequence and structure novelty using pLM‐Repeat and other tools (see section 2 for details).

**FIGURE 6:**
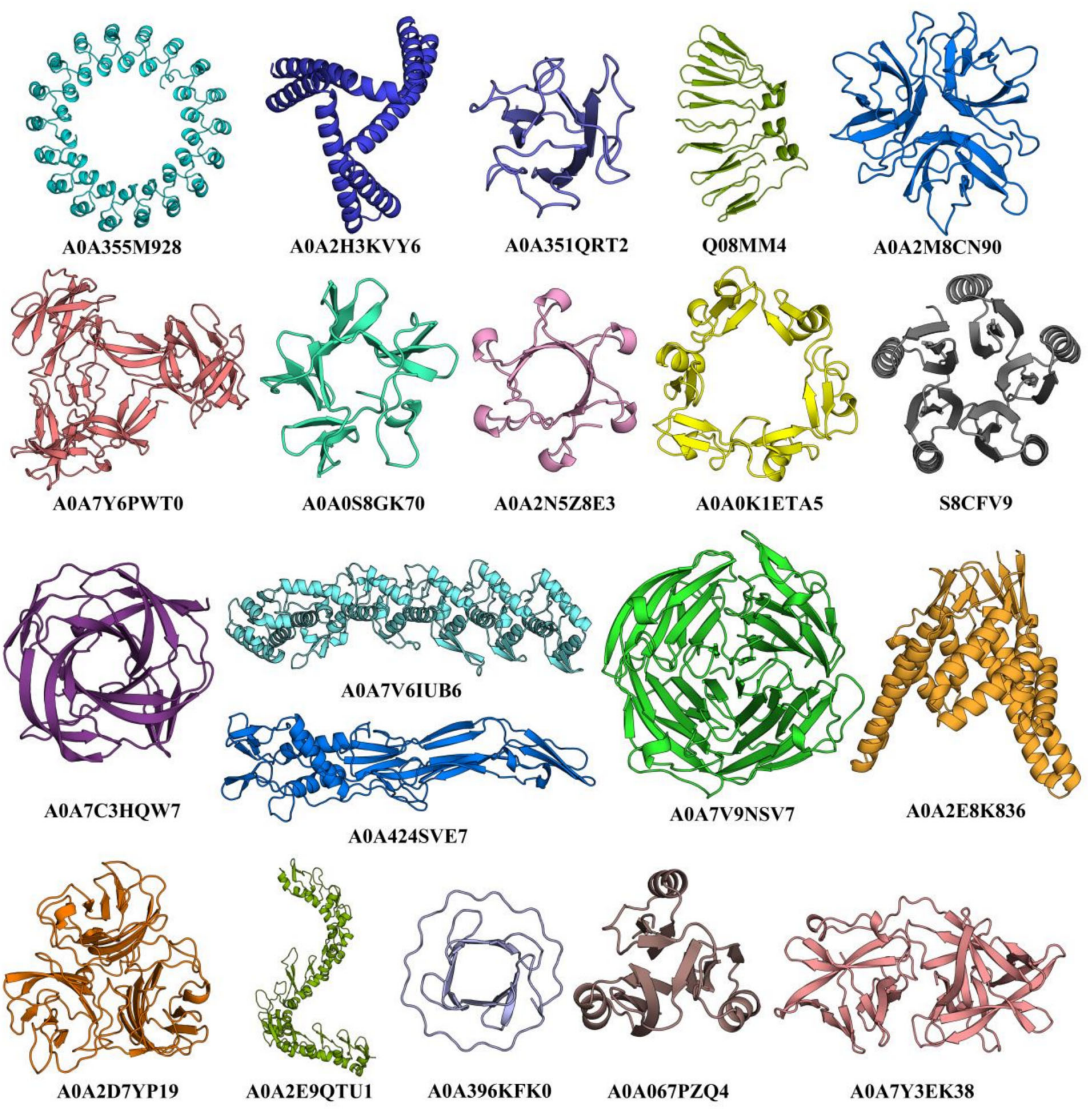
Gallery of potentially novel repeat proteins. A collection of potentially novel repeat proteins detected in the AFDB90v4.

Although the DeepRepeat model was trained to detect repeat protein domains based on prior knowledge rather than to identify *de novo* repeat patterns, some proteins predicted to contain repeat domains exhibit sequence and structure novelty compared to well‐defined repeat folds. Figure 6 shows a selection of such potentially novel repeat proteins discovered in our pipeline. One protein of significant interest (UniProt ID: A0A7C3HQW7) contains four RUs that fold into twisted long β‐hairpins (first structure in the third row in Figure 6, see also Figure S7). Interestingly, although the inner core of this structure resembles a β‐barrel, no homology to any β‐barrel could be detected using HHpred or pLM‐BLAST searches, regardless of whether the search was performed with the full‐length sequence or only the barrel region.

Another protein with a novel repeat topology is A0A424SVE7. Its RU adopts a complex structure starting with a small β‐hairpin, followed by an α‐helix and an elongated β‐hairpin composed of several separate β‐strands (second structure in the third row in Figure 6, see also Figure S8). Similarly, A0A0S8GK70 showcases a structurally unique fold, characterized by 5‐fold internal symmetry and RUs consisting of three β‐strands, where the first and last strands interact with adjacent repeats (second structure in the second row of Figure 6, also see Figure S9). A more rigorous assessment of novelty, coupled with further classification of these repetitive domains, will be crucial to advancing the field of repeat protein studies.

## 4 DISCUSSION

Sequence representations derived from protein language models (pLMs) provide new opportunities to address classical protein bioinformatics tasks, such as homology detection (Kaminski et al. 2023; Pantolini et al. 2024), classification (Chen et al. 2024), function annotation (Kroll et al. 2023), molecular engineering and design (Hie et al. 2024; Shanker et al. 2024). Here, we investigated the application of protein sequence representations for repeat pattern detection. By leveraging the sensitivity of pLM‐BLAST in remote homology detection and its ability to generate high‐quality suboptimal alignments, we developed pLM‐Repeat. For a given protein sequence, pLM‐Repeat first generates a set of self‐alignments and then applies a transitivity procedure and profile‐like stacking of embeddings, achieving high accuracy in detecting repeats, including those resulting from ancient amplification events.

Since pLM‐BLAST was designed as a universal tool that can be combined with **most residue‐wise embedders**, pLM‐Repeat is likewise compatible with residue‐wise embeddings from most protein language models. In this study, however, we selected ProtT5 based on preliminary tests with ESM‐family models such as ESM‐1b (Rives et al. 2021) and ESM‐2 (Lin et al. 2023). We found that self‐alignment matrices derived from ESM‐based embeddings tend to show noisy off‐diagonal signal, which can compromise the quality of suboptimal alignments used for repeat detection. ProtT5, in contrast, produced more robust self‐alignments, in line with its strong performance in our previous pLM‐BLAST homology detection benchmarks (Kaminski et al. 2023). We hypothesize that these differences may be related to a bias of ESM embeddings towards encoding structural information, potentially leading to similarities driven by local structural similarity rather than by homology. A more systematic and extensive comparison across recent pLMs, especially newer large models, would be a valuable direction for future extensions of pLM‐Repeat.

In principle, our workflow could also be extended to structure‐informed embeddings. We explored this prospect by feeding pLM‐Repeat with structure embeddings derived from two inverse folding models, ESM‐IF (Hsu et al. 2022) and MIF (Yang et al. 2023). Despite some successful cases where pLM‐Repeat reported correct repeats (Figure S10), the overall benchmark on the RepeatsDB dataset showed that CE‐symm significantly outperformed pLM‐Repeat using structure embeddings, with MIF representations achieving slightly more correct detections than ESM‐IF (Figure S11). This drop in performance when shifting from the ProtT5 model to inverse folding models may result from the fact that these models were developed primarily for capturing sequence‐to‐structure compatibility rather than evolutionary relationships, which were the main focus of pLM‐BLAST and pLM‐Repeat. The successful cases (Figure S10) show the potential to identify repetitive patterns directly from structure embeddings instead of coordinate information. Tailoring the upstream embedding alignment process and selecting appropriate structure representations will be key to unlocking this capability. Moreover, considering the strong performance of the recent structural repeat detection method STRPsearch, which is built upon Foldseek, the Foldseek 3Di structural representation could be a promising avenue for repeat detection. The 3Di alphabet encodes each residue together with its local structural environment into a compact letter, substantially accelerating structural search while retaining high sensitivity. Its low‐dimensional “structure sequence” format also makes it directly compatible with existing sequence‐based repeat‐detection algorithms, allowing it to benefit from mature components such as statistical scoring and evaluation schemes.

To facilitate the application of pLM‐Repeat to large databases such as AFDB, we trained DeepRepeat, a deep learning model that serves as a fast knowledge‐based pre‐filter to identify proteins that have patterns similar to well‐characterized repeat domains. Instead of relying on curated MSA‐based features or physico‐chemical characteristics of amino acids such as the Tally classifier (Perovic et al. 2020; Richard et al. 2016), DeepRepeat uses only raw pLM embeddings output from pLMs for both training and prediction. In this work, we used DeepRepeat to scan protein sequences with low “functional brightness” annotated in AFDB90v4 (Durairaj et al. 2023) followed by sequence and structural analysis, as these proteins have predicted structures in AFDB for easy manual verification and validation of whether the detected proteins actually contain repeats. This pipeline led to the discovery of a number of novel repetitive domains (Figure 6), some of which were particularly intriguing, such as the 4‐copy β‐barrel‐like domain composed of twisted β‐hairpins (Figure S7). These sequence‐based analyses are complementary to the recently released TED database performing symmetry detection directly on the AFDB models using SymD, a tool for identification of internally symmetric protein structures (Lau et al. 2024). Prompted by these results, we envision further enhancements to the DeepRepeat model that would enable it to detect *de novo* repeat patterns encoded in sequence embeddings and even report repeat regions. This capability has been implemented at the structural level in DeepSymmetry, which detects structural repeats and density maps using 3D convolutional networks (Pagès and Grudinin 2019), suggesting that such a generalization of the DeepRepeat model is feasible. Moreover, the accuracy of the model may benefit from a training scheme based on individual domains given the recent advancements in domain prediction for both sequences and structures (Iovino et al. 2024; Wells et al. 2024).

A limitation of pLM‐Repeat is the lack of rigorous statistical evaluation, beyond the alignment score provided by pLM‐BLAST. Traditional statistical frameworks, such as the extreme value distribution theory used in HHrepID, may not be appropriate for protein embeddings as shuffling residue embeddings would disrupt the context‐dependent nature of pLM representations. The lack of a robust statistical framework can pose challenges to the pLM‐Repeat procedure in certain steps. For example, an observed problem is the redundancy of detected traces that are only a few residues apart from each other. While HHrepID severely penalizes such shifted alignments, in pLM‐Repeat they may still pass the suboptimal alignments selection step if they exceed the given threshold (see Figure S12 and accompanying text for details). Another shortcoming is the output of multiple alignments of detected repeat instances. Currently, pLM‐Repeat generates the alignment of all detected repeats by simply concatenating pairwise alignments of each detected repeat to the reference repeat (Figure 1), resulting in alignments that are not optimal (e.g., the output multiple alignment in Figure 1). Recent work using pLM embeddings for MSA construction may provide a possible solution. However, most of these recently developed strategies, such as vcMSA (McWhite et al. 2023) and PEbA (Iovino and Ye 2024), rely on clustering and ordering of residue embeddings and are designed to process gapless sequences as input. Consequently, they fail to take advantage of the valuable pairwise alignment obtained during the self‐alignment process.

In sum, by introducing pLM‐Repeat and benchmarking it systematically against the MSA‐based HHrepID, we **aim to** establish a fast yet sensitive framework, thereby expanding the toolkit of repeat detection methods. Selecting the appropriate tool **within this toolkit** remains a central consideration in repeat analysis. Knowledge‐driven algorithms are highly effective for well‐characterized repeat families, whereas *de novo* strategies offer the advantage of identifying previously unannotated repeat proteins. pLM‐Repeat provides competitive sensitivity without relying on MSAs, substantially reducing computational time and showing particular strength on specific protein folds such as β‐propellers. HHrepID, however, continues to excel at robustly detecting highly divergent repeats due to its mature statistical framework, making it a reliable option for performing detailed downstream analyses. One general limitation of embedding‐based approaches is the computational cost associated with generating and storing residue‐level embeddings, which may hinder their use in large‐scale scans. In such settings, lower‐complexity strategies, such as seed‐extension–based approaches exemplified by the recently proposed DetectRepeat, can offer a more practical balance between speed and sensitivity (Cho and Wright 2025). Meanwhile, as structural information becomes increasingly available, particularly with the release of AFDB, structural repeat annotation and prediction represent an attractive direction for uncovering additional “dark” repeat proteins. Tools such as STRPsearch will become even more powerful when combined with sequence‐based methods that capture evolutionary signals. Overall, effective repeat detection requires matching the algorithm, data modality, and analysis strategy to the biological question. Leveraging complementary approaches, rather than relying on a single paradigm, will ultimately provide the most comprehensive and accurate characterization of repeat proteins.

## AUTHOR CONTRIBUTIONS

**Kaiyu Qiu:** Conceptualization; methodology; software; data curation; formal analysis; investigation; project administration; writing – original draft; writing – review and editing; visualization. **Andrei N. Lupas:** Conceptualization; supervision; writing – review and editing; funding acquisition. **Stanislaw Dunin‐Horkawicz:** Conceptualization; methodology; writing – original draft; writing – review and editing; supervision.

## Supporting information


**Data S1.** Supporting Information.

**Data S2.** Supporting Information table.

## Data Availability

Source codes of pLM‐Repeat are available on the GitHub repository https://github.com/KYQiu21/plmrepeat/. A Colab notebook for running pLM‐Repeat in an interactive way is available at https://colab.research.google.com/drive/1ouBwciiXy7HPnddut15JAGAmREWaqaZ7. The benchmark dataset, together with the trained model weight, and the generated dataset are available on the Zenodo repository https://zenodo.org/records/14245453.
