# Supplementary material for: pLM‐Repeat: Exploiting the sequence representations of protein language models for sensitive repeat detection
Source: Protein Sci. 2026 Apr 7;35(5):e70541. doi: 10.1002/pro.70541 (PMC13055192; doi:10.1002/pro.70541)
Supplement: Supplementary file 1 — Data S1. Supporting Information. [file PRO-35-e70541-s001.pdf]

pLM-Repeat: exploiting the sequence representations of protein language models for sensitive repeat detection

Kaiyu Qiu[1]*, Andrei N. Lupas[1], Stanislaw Dunin-Horkawicz[1,2]

[1] Department of Protein Evolution, Max Planck Institute for Biology Tübingen, Tübingen, Germany

[2] Institute of Evolutionary Biology, Faculty of Biology, Biological and Chemical Research Centre, University of Warsaw, Warsaw, Poland

* Correspondence: kaiyu.qiu@tuebingen.mpg.de

Supplementary Figures

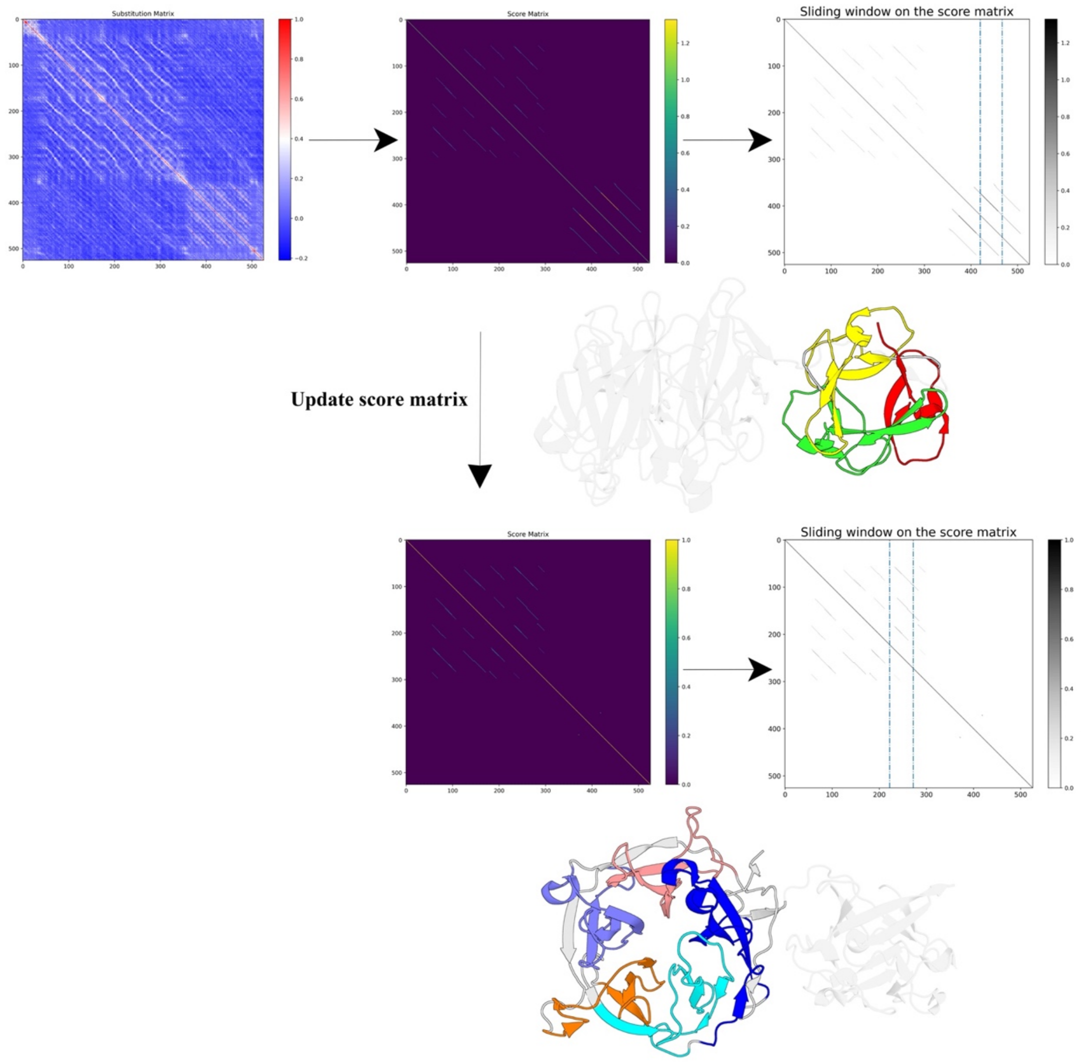

Supplementary Figure 1: pLM-Repeat analysis on the domain 3VT1_A, which contains two types of repeats. 5 propeller repeats and 3 trefoil repeats were reported.

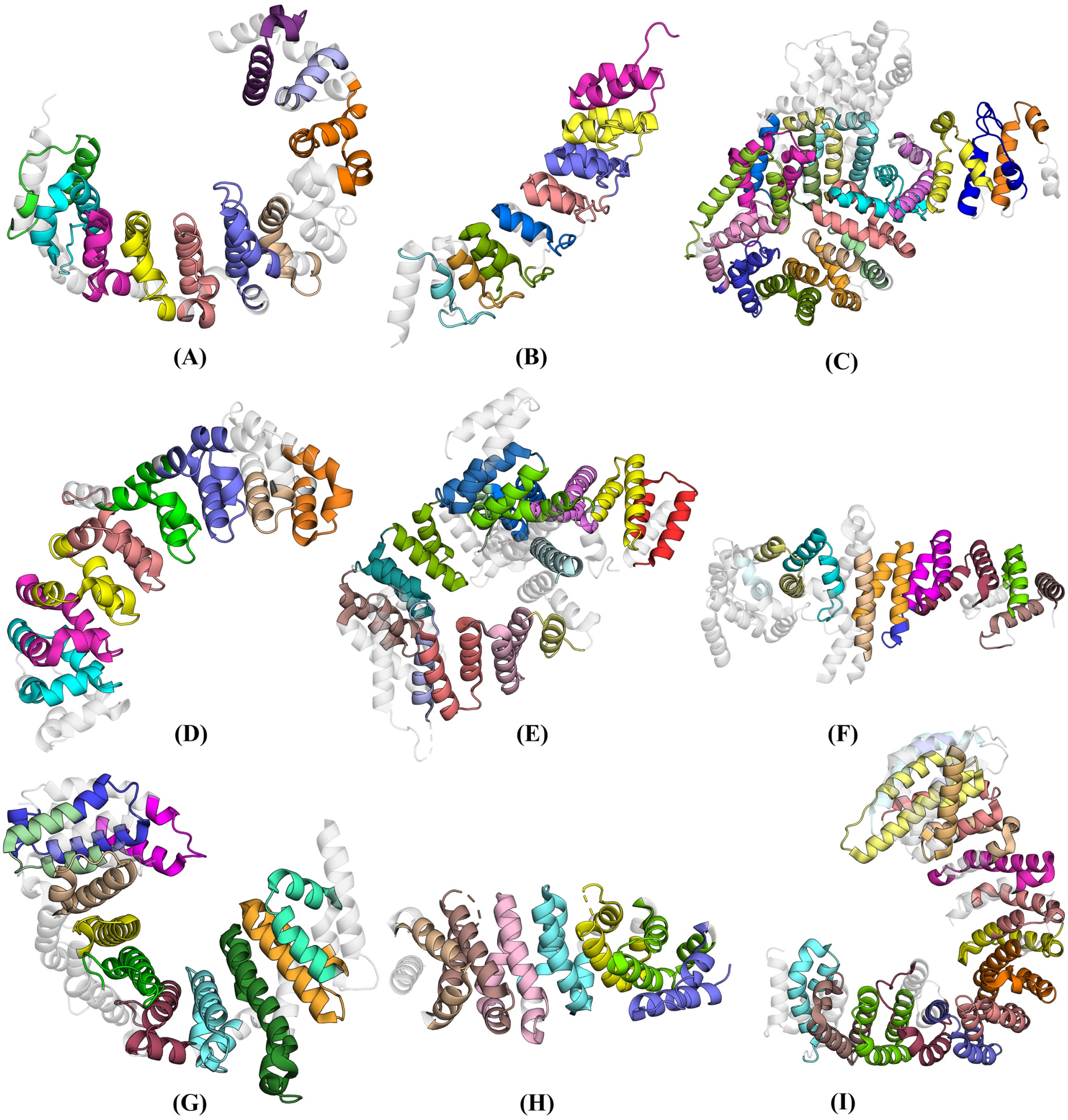

Supplementary Figure 2: A selection of α-solenoid domains correctly detected by the pLM-Repeat method. Protein structures are colored according to repeat ranges. (A) 4A3V_A, (B) 6BY9_A, (C) 6QDK_A, (D) 3V71_A, (E) 5NNP_A, (F) 3TJ1_A, (G) 4GMO_A, (H) 3T7U_A, and (I) 2QNA_A.

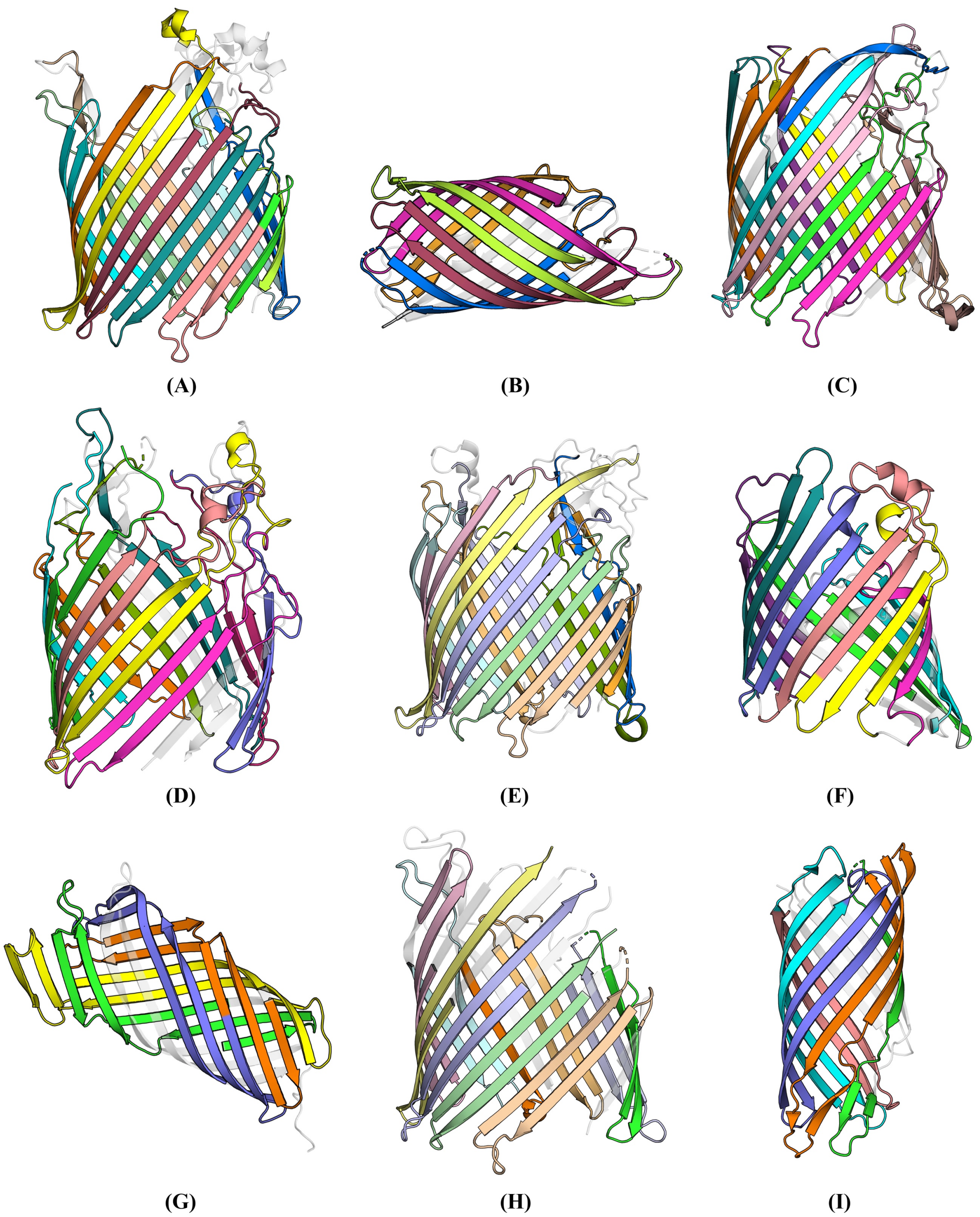

Supplementary Figure 3: A selection of β-barrel domains correctly detected by the pLM-Repeat method. Protein structures are colored according to repeat ranges. (A) 6E4V_A, (B) 2QOM_A, (C) 3QLB_A, (D) 1FEP_A, (E) 3ODW_A, (F) 6ENE_A, (G) 2X4M_A, (H) 3EFM_A, and (I) 4MEE_A.

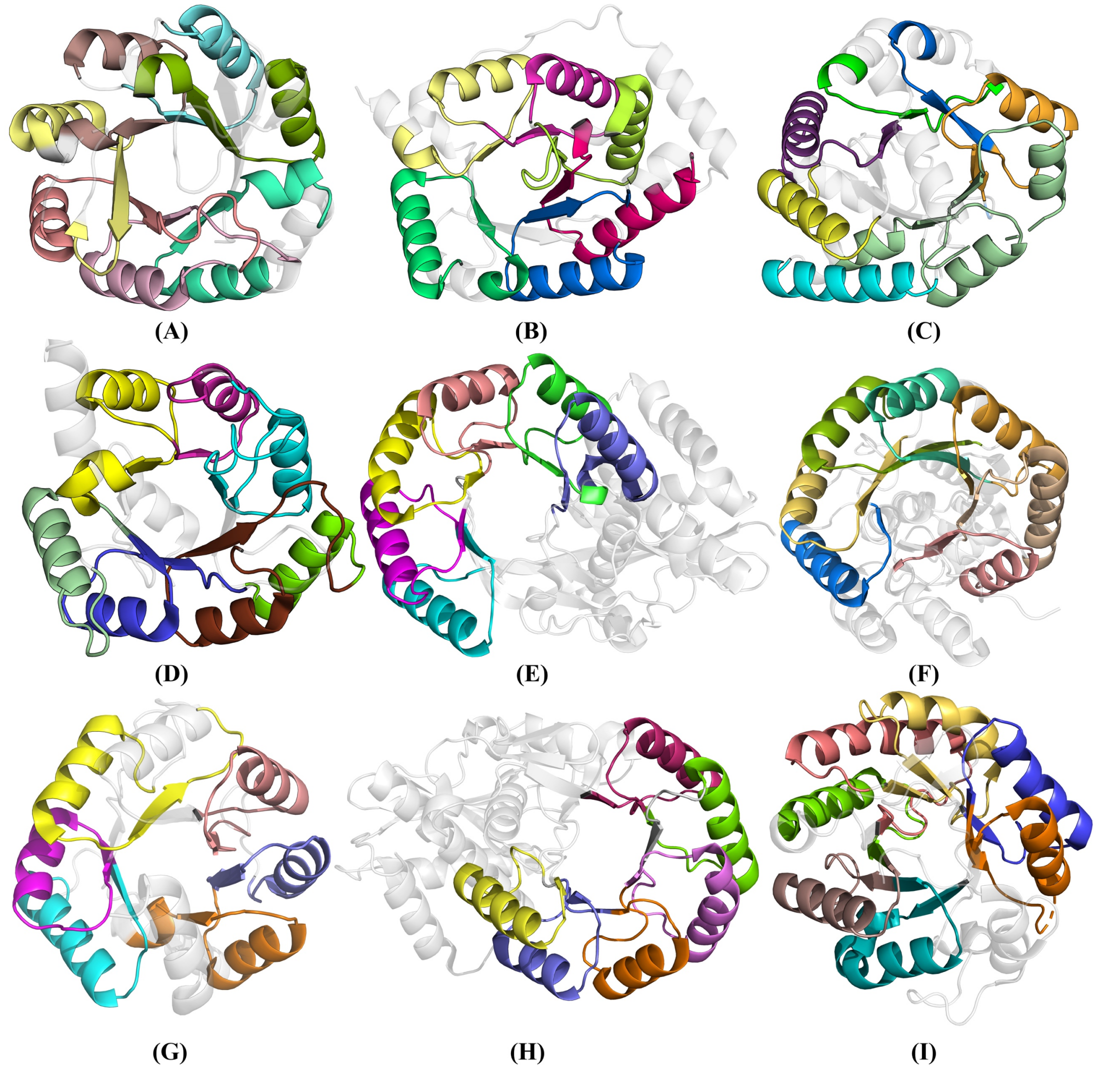

Supplementary Figure 4: A selection of TIM-barrel domains correctly detected by the pLM-Repeat method when the self-alignment score threshold was lowered to 0.25. Protein structures are colored according to repeat ranges. (A) 4W9T_A, (B) 3OA3_A, (C) 1A50_A, (D) 1YXY_A, (E) 2PGW_A, (F) 1NVM_A, (G) 3IGS_A, (H) 2QGY_A, and (I) 3JUG_A.

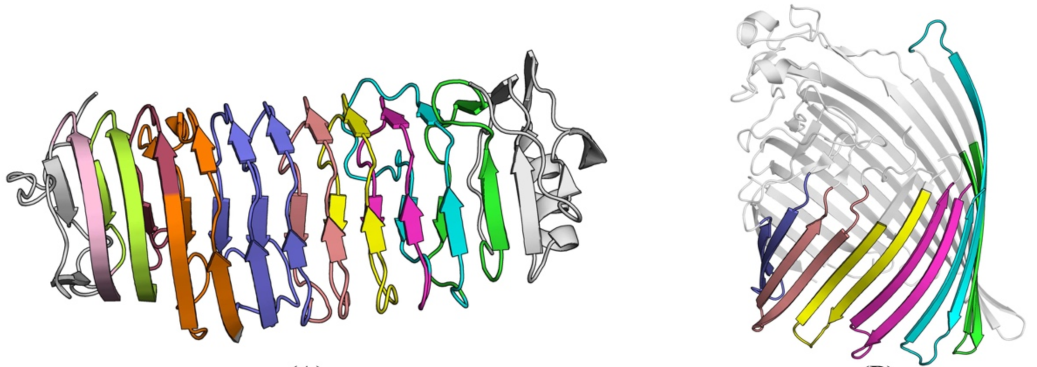

(A) **(B)**

Supplementary Figure 5: Performance of HHrepID on the domain (A) 7C7D_A and (B) 5NEC_A. Structures are colored according to the identified repeat regions. HHrepID fails to capture any repeat on the domain 2X19_B and 3NAV_A shown in the main Figure 3.

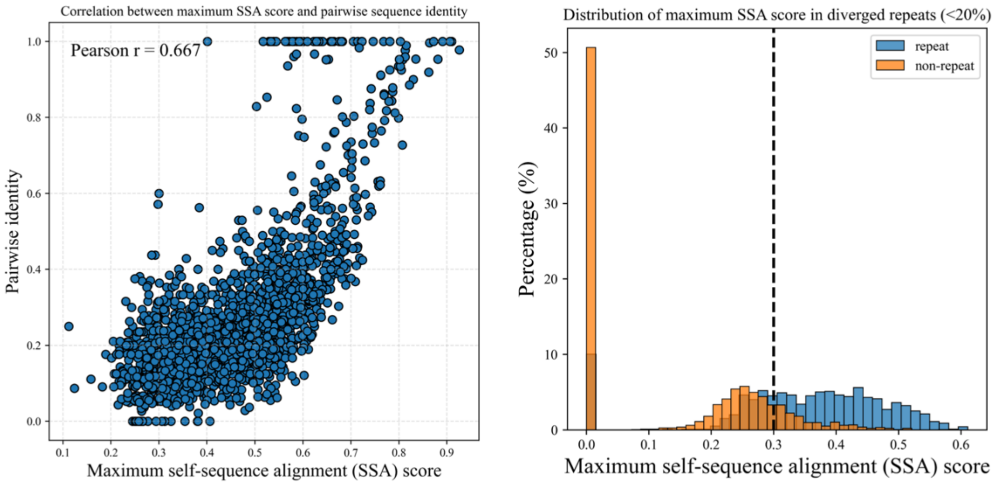

(A) **(B)**

Supplementary Figure 6: Distribution of self-alignment scores. In both panels, the pLM-BLAST self-alignment score for each sequence is the maximum off-diagonal score. (A) Each point corresponds to one RepeatsDB benchmark entry; the x-axis shows the maximum off-diagonal score and the y-axis shows the sequence identity of the corresponding aligned region. (B) Score distributions for diverged repeats (mean all-vs-all RU–RU identity <20% per entry, blue) and non-repeat proteins from the negative benchmark set (orange; see Methods). The default score threshold (0.3) is indicated by the vertical dashed line.

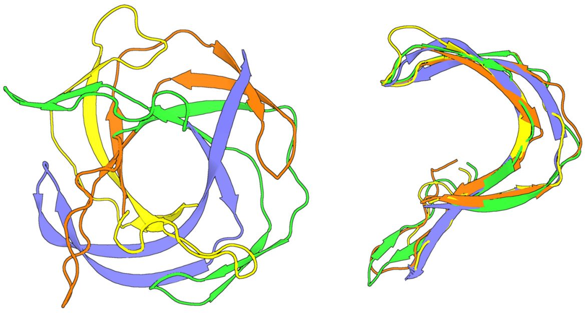

Supplementary Figure 7: The structure of domain A0A7C3HQW7 colored by four manually curated structural repeats.

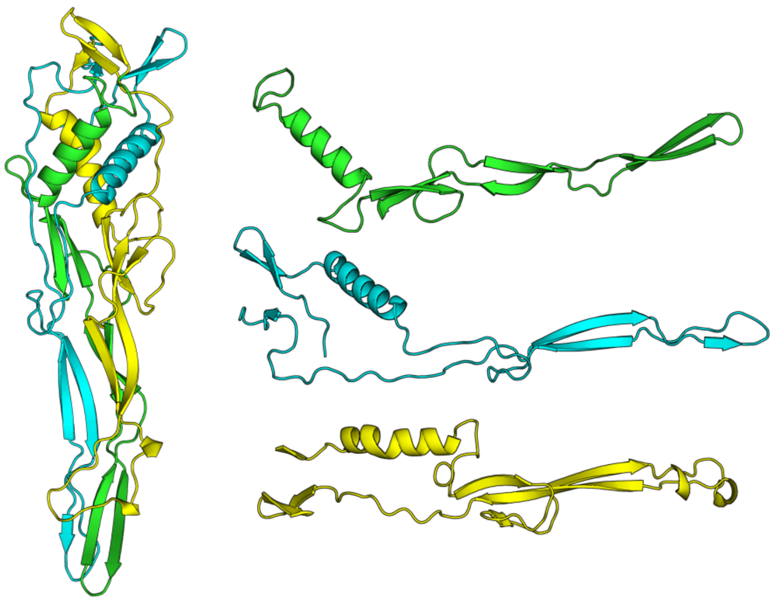

Supplementary Figure 8: The structure of domain A0A424SVE7 colored by three manually curated structural repeats.

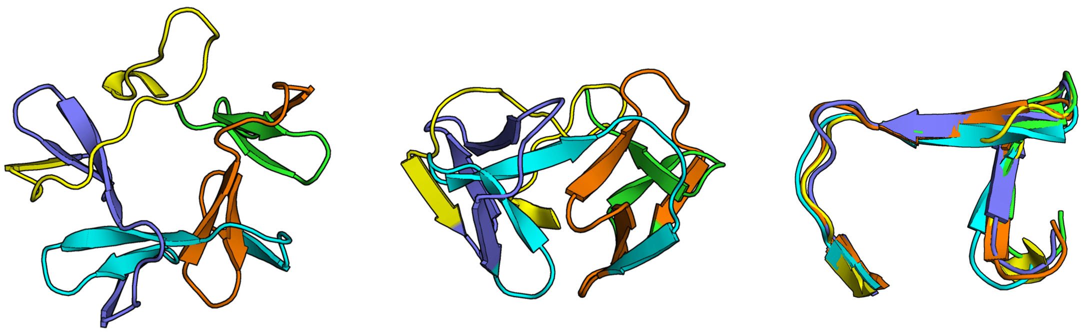

Supplementary Figure 9: The structure of domain A0A0S8GK70 colored by five manually curated structural repeats.

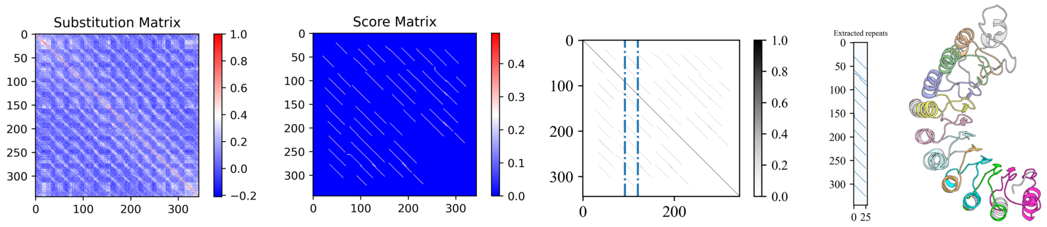

Supplementary Figure 10: pLM-Repeat intermediate outputs and detected repeats with ESM-IF structural embeddings as inputs on the domain 1K5D_C.

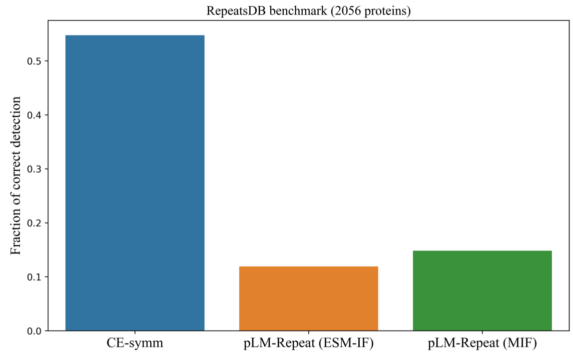

(A)

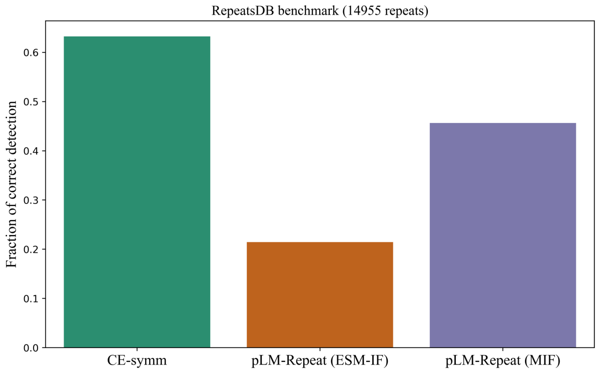

(B)

Supplementary Figure 11: Performance of pLM-Repeat equipped with structure embeddings (from two inverse folding models, ESM-IF and MIF) at the protein (A) and repeat (B) level, together with the state-of-the-art structure-based repeat detection software CE-symm.

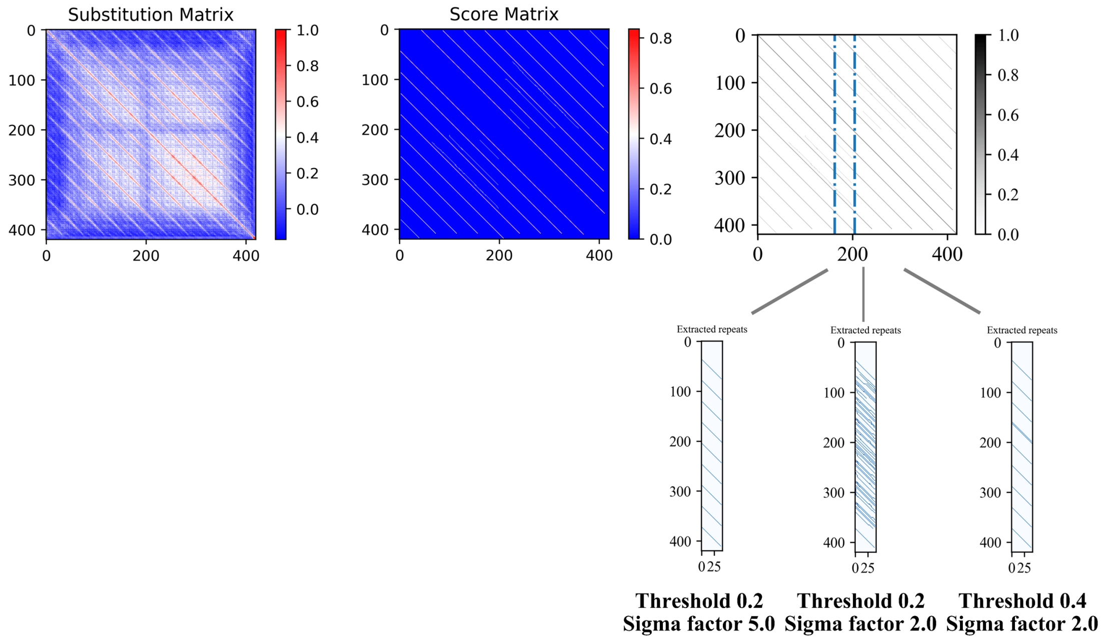

Supplementary Figure 12: The repeat extraction step is influenced by several factors, as in the example case of the domain PDB:4RV1_A. Substitution matrix, score matrix and sliding window are generated correctly and show accurate periodicity. However, a Sigma factor of 2.0 and an extraction score threshold of 0.2 result in redundant detections. One solution to this problem is to increase the default sigma factor in pLM-BLAST. The default sigma factor of 2.0 indicates that subpaths reported by pLM-BLAST must have a minimum score of at least two times the average standard deviation of the substitution matrix. Increasing this parameter will therefore make the selection of subpaths more stringent. Alternatively, a similar effect can be achieved by increasing the pLM-BLAST score threshold of the collected suboptimal self-alignments.

Supplementary Text:

A. Parameters of pLM-Repeat

(1) Input:

Protein sequence: the query sequence of a length L to be analyzed

Protein embedding: the residue-wise embedding with a shape (L, N_{dim}) of a protein sequence

Output directory: the output directory to store results

(2) pLM-BLAST-related (two rounds of pLM-BLAST are conducted during a pLM-Repeat job, one for identifying suboptimal self-alignments, one for scanning for repeat instances):

Window length: the length of the moving sliding window used to detect local alignments within the paths determined by the traceback procedure (default: 15)

Minimum span length: the minimum length of matches returned during the traceback step, cannot be less than the window length (default: 15)

Self-sequence alignment score: the minimum score for each reported alignment in the first round of pLM-BLAST to conduct the self-sequence alignment, the score is derived from the cosine similarity substitution matrix based on the average of all aligned pairs (default: 0.3)

Sigma factor: the cutoff at which the background signal is discarded when searching for significant local alignments during self-alignments, the higher the Sigma factor, the stricter the algorithm (default: 2.0)

Scan self-sequence alignment score: the minimum score for each reported alignment in the second round of pLM-BLAST to scan the representative repeat against the full-length sequence for extracting repeat units (default: 0.2)

Scan sigma factor: the cutoff at which the background signal is discarded when searching for significant local alignments during repeat instance extraction (default: 2.0)

Weighted embedding: whether to use weighted representative repeat embedding based on score matrix (default: True)

(3) pLM-Repeat-related:

Transitivity: whether to generate transitive traces or not (default: True)

Transitive trace score: the minimum score threshold of transitive traces to be considered in the following step (default: 0.0)

Repeat overlap: the maximum overlap fraction of two adjacent detected repeats before refining (default: 0.3)

Metrics: the evaluation metrics used to determine the reported repeats from a set of possibilities (default: 'repeat_total_len')

Draw: whether to draw intermediate outputs (i.e. score matrix, substitution matrix, etc.) or not (default: True)

B. pLM-Repeat analysis results of examples shown in the manuscript:

Each example includes the cosine similarity substitution matrix, the derived score matrix, the determined representative repeat window, and the concatenated alignment.

2X19 (Figure 3A)

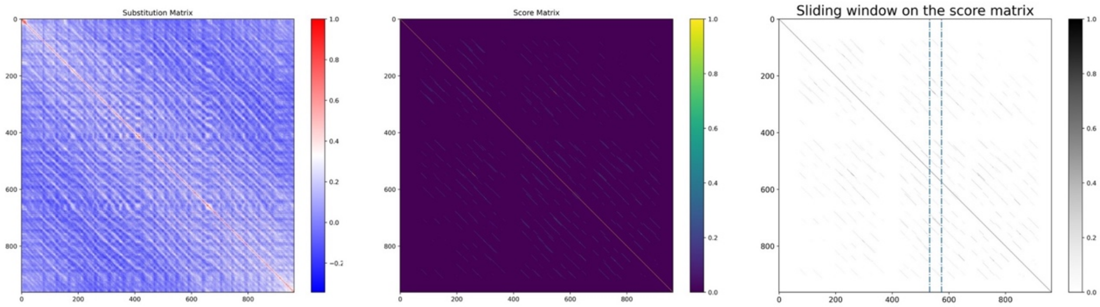


```
>representative
LV-------L-H-ALG-----N-P-E-----L--S----V-------S---SVSTLKKI-CR-ECK-Y-D-L-P-P----Y---AA-N--IV---AVSQ-----
>repeat 1|score=0.3277216|64-93
LL-------------Q-----P--D-KVPE-I--Q----Y-------F---GASALHIKI-S-RYW-S-D-I--------------------------------
>repeat 2|score=0.37933|107-141
QI-------T-R-FAS-----GS-K-I-----V-L----T-------R---LCVALASL-ALS-MM-PD--A------------WP-C----------------
>repeat 3|score=0.33728862|207-235
LL-------------Q-----Q-P-S-----S--PSCVRQ-------K---VLKCFSSW-VQ-LE-V-P-----------------------------------
>repeat 4|score=0.51396424|244-279
AA-------F-A-ALQ-----D-S-E-----L--F----D-------S---SVEAIVNA-IS-QPDA-Q-R-YV-N----T---L------------------
>repeat 5|score=0.4436488|294-341
-----------Q-AVQ-----N-G--DME--T--S----H-------G---ICRIAVAL-GE-NHS-R-A-L-L-D----QVEHWQ-S--FLALVNMIMFC---
>repeat 6|score=0.31930527|341-371
--------------TG-----I-P-GHYPV--N------ETTSS---L---TLTFWYTL-QD-DI--------------------------------------
>repeat 7|score=0.46171904|415-451
-------------------------EKE------------QFRIYRVD---ISDTLMYV-YE-MLG-A-E-L-LS-----N---L------Y---DKLG-----
>repeat 8|score=0.50799286|452-489
LL--------T-----SSE---EP-YSWQ---H-T----E-------A---LLYGFQSI-AE-TID-V-N-Y-S-D----V---VP-----------------
>repeat 9|score=0.5336225|489-515
GL-------I-G-LIPRISIS-N-V-Q-----L-A----D-------T---VMFTIGAL-SE-WLA--D-H-P--V---------------------------
>repeat 10|score=0.63944244|515-568
MINSVLPLVL-H-ALG-----N-P-E-----L--S----V-------S---SVSTLKKI-CR-ECK-Y-D-L-P-P----Y---AA-N---------------
>repeat 11|score=0.5371373|575-623
VL-------M-K-QIH-----K-T-S-----Q--C----M-------W---LMQALGFL-LS-ALQ-VE-E-IL-K----N---LHS---LI---SPYIQQLEK
>repeat 12|score=0.31707162|626-651
-----------E-I-P-----N-P-S-----N--K-----L------AIVHILGLLSNL-FT-TL--------------------------------------
>repeat 13|score=0.63604575|690-729
VL--------S-KWL-N----D-A--Q----V--V----E-------A---VCAIFEKS-VK-TLL-D-D-F-A-P----M---VP-Q--LC---E--------
>repeat 14|score=0.5102122|733-770
-------------MYS-----T-I--P----Q---A-----------S---ALDLTRQL-VH-IFA-H-E-P----AHFPP---IEA---LF---LL-------
>repeat 15|score=0.4405178|777-816
LF--------Q-----Q------GP-RDHPDI--V----D-------S---FMQLLAQA-LK-RKP-D--L-FL-CE----R------LDV----K--------
>repeat 16|score=0.34688747|823-848
LA------------LK-----F-P-EA-----PTV----K-------A---SCGFFTEL-LP-RC--------------------------------------
>repeat 17|score=0.42187548|871-907
AI-------------GG-----Q-A-SRS--L--M----D-------C---FADILFAL-NK-HCF-S-L-L-S-M----W---IK-E--A------------
```

7C7D (Figure 3B)

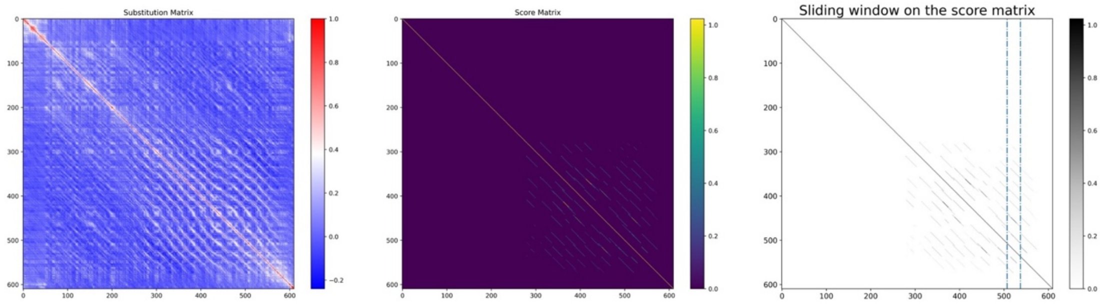


```
>representative
GG--Q--G--H---A-VK-NVT--VD-G-AA-VK-NTGT-V-V--V-QAE--AP---
>repeat1|score=0.18075006|250-267
-GP----LS--G----VQ-NVT--LQ-G-A-GS-W----------------------
>repeat2|score=0.31108323|277-295
-DQT-DS--------A-G-HVH--LK-D-FA-VI-G---------------------
>repeat3|score=0.43550572|309-333
G-S-----------L-GP-GSS--VS-G-MW-IQ-HVKV--G---L-WL--------
>repeat4|score=0.5709188|333-356
T-G-T-----------ND-DLV--VE-N-NR-IL-DTTA-D-G--L-N---------
```

```
>repeat5|score=0.57309157|356-381
G-T-------------AK-NVT--VR-D-NF-LR-NQGD--DA--L-AM--------
>repeat6|score=0.54525864|382-407
SLY-----A--P----DT-DCR--FE-N-NT-IT-QPNLA-N-G--I----------
>repeat7|score=0.5384166|410-437
G----------------T-DIT--VK-G-NL-IS-DTNA-L-GSGI-AIS--NQ---
>repeat8|score=0.5358004|436-461
K-FA-E----PFHP-LAG-TIT--VD-G-NT-LV-RTG-------------------
>repeat9|score=0.60729325|477-502
SYD-----S-A---I-EA-RVD--IT-D-TT-IT-DSPY-S-A--F-----------
>repeat10|score=0.7803913|506-536
G-G--Q-G--H---A-VK-NVT--VD-G-AA-VK-NTGT-V-V--V-QAE--A----
>repeat11|score=0.4846747|536-562
-----------G------E-AT--FR-N-VT-AT-GTGA-A-G--I-YN-CP--FPS
>repeat12|score=0.27116436|558-579
--------GS-G---TFT----VTD-GGGNS----G--W--------D---------
```

5NEC (Figure 3C)

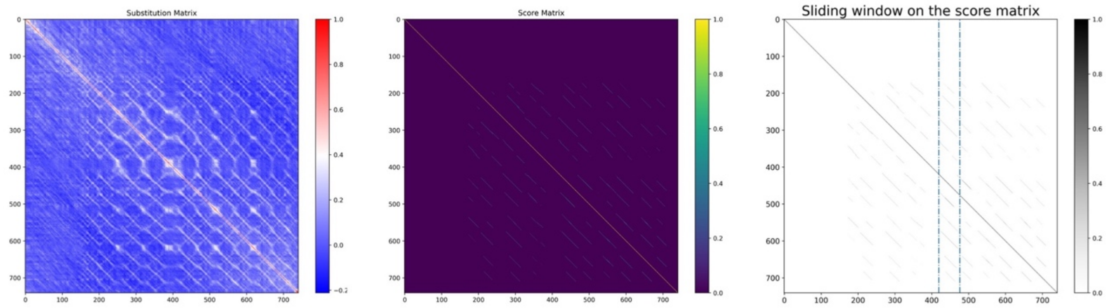


```
>representative
TR-------------K-Y----A--PL--N-T-VGTTKAIYAFDT--IDLNE-Q-WQVNIGARFDS-F-E--T-T-A--K-N-H-G--V-R-PA-TKLSDK
>repeat1|score=0.3801994|197-239
AN--------------V-A----G-RD---EV-DVSRWGVAPSLT--FGLGS-P-TRVTVSHYHLE-S-D--D-T-------------------------
>repeat2|score=0.5124482|261-317
Y-G------------L-T----G--RD--F-Q-KSRIDTSTITVE--HDLTD-S-LTIRNTSRYGN-S-H--Q-D-Y--L-W-T-Q--P-D---------
>repeat3|score=0.26667833|324-356
NGSVWR---------R-Q----N--NR--V-S-TTTTAVNQTDLFGEFYLGG-----------------------------------------------
>repeat4|score=0.6885057|407-471
-NPNPHDPWNGSITRK-Y----A--PL--N-T-VGTTKAIYAFDT--IDLNE-Q-WQVNIGARFDS-F-E--T-T-A--K-N-H-G--V-R-PA-T-----
>repeat5|score=0.44753775|471-517
-------------------------KL--S-D-KSSFWNWQAGLV--WKPVP-N-GSIYASYATSA--TP---PG--S-M-LD---NG-D-T-S-------
>repeat6|score=0.30962577|526-556
---------------NN------L------E-P-EETTNYELGTK--WAFFNE-RLELSAAI--------------------------------------
>repeat7|score=0.48458055|563-619
TR-------------IL-VANQ-TY--DN-AG-QSRVDGVELSAS--GKLTE-K-WKVFAGYSYLD-S-E--L-V-DA---G-K-A--G-R-NG-------
>repeat8|score=0.4553247|639-674
------------------------------------SF--SLWTT--YDIFP-K-TTIGGGAFYVD-K--V--Y-G-D--V-G-NT--V-Y----------
>repeat9|score=0.41559586|683-719
----------------------------------------MA--S--YKLSK-N-VDFQLNVQNVFD---KK---Y-FD-K-A-Y-A-A-H-Y-ASQ----
```

3NAV (Figure 3D)

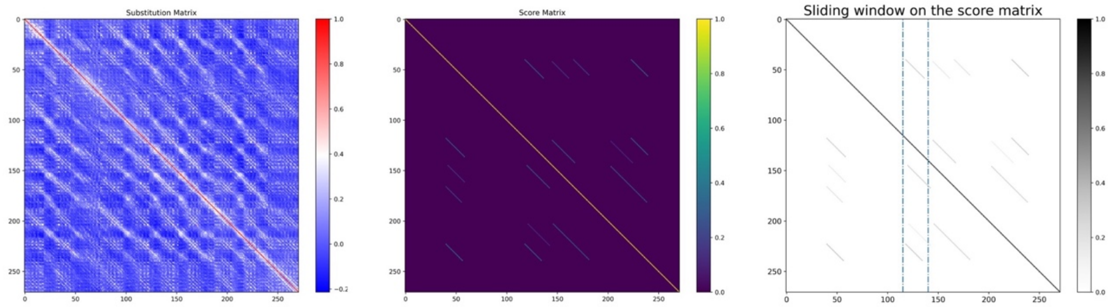


```
>representative
DF-YQ-RCQ-KAG-VD--S-V--L-I-A-DV-P-T-NESQ
>repeat1|score=0.48873338|37-58
AI-MQ-TLI-DAG-AD--A-L--E-L-G-MP-F-S-----
>repeat2|score=0.25580636|89-111
----Q-I-RA-RNP-ETP--IGL-L-MYANLV-Y-A----
>repeat3|score=0.7420481|111-134
DF-YQ-RCQ-KAG-VD--S-V--L-I-A-DV---------
>repeat4|score=0.47599897|140-160
PF-VA-AAE-KFG-IQ--P-I--F-I-A-PP-T-------
>repeat5|score=0.24204922|163-180
ETL-R-AVAQ-LG-K-G--YT--YL-L-------------
>repeat6|score=0.3127343|197-217
AL-LE-RLQ-QFD-AP--P-A--L-L-G-FG-I-------
>repeat7|score=0.4217853|220-241
AQV-KQ-AI-EAG-AA--G-A--I-S-G-SA-V-V-----
```

1W6S (Figure 4)

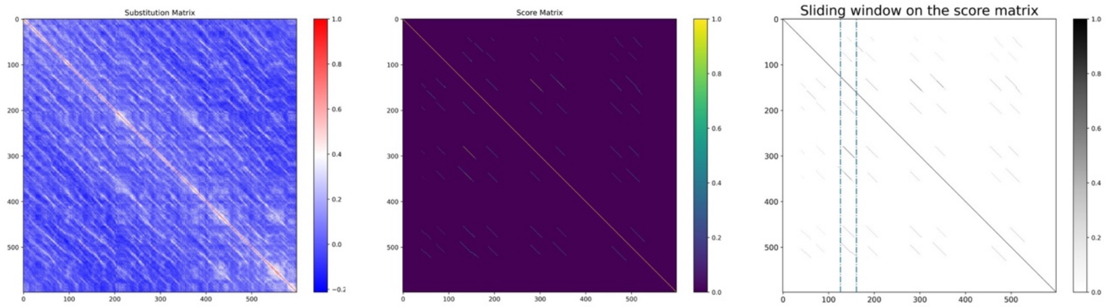


```
>representative
TQLDGNVAALNAETG-ETVWKVENSD-IK-V-GSTLTIA
>repeat 1|score=0.4084017|68-96
TSFPNNTFALGLDDPGTILWQDKPK-QN-P---------
>repeat 2|score=0.8174701|126-160
TQLDGNVAALNAETG-ETVWKVENSD-IK-V-GSTLTI-
>repeat 3|score=0.53519267|177-209
LGVRGYLTAYDVKTG-EQVWRAYATG-PD-K-DLL--L-
>repeat 4|score=0.6201753|273-305
NKWTMTIFGRDADTG-EAKFGYQKTP-HD-E-WDYA---
>repeat 5|score=0.5050528|328-351
PDRNGIVYTLDRTDG-ALVSANKL---------------
>repeat 6|score=0.52561426|448-480
YEGLGQIKAYNAITG-DYKWEKMERF-AV--WGGTM---
>repeat 7|score=0.5577061|489-512
GTLDGYLKARDSDTG-DLLWKFKI---------------
```

C. DeepRepeat implementation details:

DeepRepeat uses a light-weight attention-based architecture trained with 2056 and 8710 positive and negative examples, respectively (see the main text for the details of dataset creation). For each protein, we start from per-residue embeddings $x \in \mathbb{R}^{B \times L \times D}$ (with embedding dimension D = 1024) and first reshape them to B×D×L to apply 1D convolutions along the sequence axis. Two parallel convolutional layers with kernel size 9 and padding 4 are used: a feature convolution that produces a contextualized feature map and an *attention* convolution that produces unnormalized attention scores. The attention scores are masked such that padded positions (introduced when batching sequences of different lengths) are set to a large negative value and are therefore ignored, and a *softmax* over the sequence dimension yields normalized attention weights. The feature map is then aggregated in two complementary ways: (i) an attention-weighted sum over positions and (ii) a max-pooling over positions. The resulting two D-dimensional vectors are concatenated into a 2D-dimensional representation, which is passed through a small feed-forward network consisting of a linear layer with 32 hidden units, ReLU activation, dropout and batch normalization. A final linear layer maps this 32-dimensional representation to the two output logits, and a *softmax* produces class probabilities. Dropout is applied both after the feature convolution and in the classification head to regularize the model. DeepRepeat was trained on the NVIDIA A100 GPU (40GB). The best model was saved at epoch 198 (selected by early stopping) with a learning rate of 10^{-6}. After obtaining the model, the prediction on a protein of 500 amino acids takes around 0.0001s on the NVIDIA A100 GPU (40GB).

Supplementary Table 1:

Fold Class	pLM-Repeat (0.3)	pLM-Repeat (0.25)
β-Solenoid	71	67
α/β-Solenoid	104	103
α-Solenoid	336	**344**
β-Hairpins	17	16
Box	40	**43**
TIM-Barrel	45	**107**
β-barrel/hairpins	41	**45**
β-Propeller	259	**274**
α/β-Prism	22	**25**
α-Barrel	24	23
α/β-Trefoil	38	36
Aligned-prism	7	6
α-Beads	7	7
β-Beads	18	16
α/β-Beads	18	18
β-Sandwich-beads	16	15
α/β-Sandwich	12	**14**

Number of correctly detected proteins in the RepeatsDB dataset based on fold classes in pLM-Repeat with a self-alignment score threshold of 0.25 and 0.3 (as in Table 1 in the main text).

Supplementary Table 2:

Fold Class	pLM-Repeat	HHrepID (MSA)	HHrepID (single)	RADAR
LRR (1816)	132 (7.3%)	**165** (9.1%)	85 (4.7%)	153 (8.4%)
LDL-receptor (21)	16 (76.2%)	**17** (81.0%)	12 (57.1%)	15 (71.4%)
ANK (139)	114 (82.0%)	**118** (84.8%)	96 (69.1%)	97 (69.8%)
HEAT (201)	**82** (40.8%)	43 (21.4%)	9 (4.5%)	38 (18.9%)
TPR (111)	**84** (75.7%)	67 (60.4%)	31 (27.9%)	57 (51.4%)
WD (139)	**110** (79.1%)	97 (69.8%)	63 (45.3%)	61 (43.9%)
LbH (77)	47 (61.0%)	**60** (77.9%)	24 (31.2%)	40 (51.9%)
PFTA (14)	**6** (42.9%)	3 (21.4%)	1 (7.1%)	1 (7.1%)
Hemopexin (11)	**11** (100.0%)	**11** (100.0%)	7 (63.6%)	4 (36.4%)
Pumilio (12)	**11** (91.7%)	**11** (91.7%)	7 (58.3%)	0 (0.0%)
Kelch (12)	**12** (100.0%)	**12** (100.0%)	11 (91.7%)	0 (0.0%)
ARM (36)	**29** (80.6%)	28 (77.8%)	15 (41.7%)	20 (55.6%)
PbH1 (32)	14 (43.8%)	**17** (53.1%)	6 (18.8%)	5 (15.6%)
PFTB (6)	**6** (100.0%)	**6** (100.0%)	2 (33.3%)	3 (33.3%)

Number of correctly detected proteins in the DbStRiPs dataset by fold class for pLM-Repeat, HHrepID (MSA), HHrepID (Single), and RADAR at default threshold settings.

Supplementary Table 3:

Evaluation	pLM-Repeat	HHrepID (MSA)	HHrepID (single)	RADAR
Positive domains (2611)	1721 (65.9%)	**1746** (66.9%)	1013 (38.8%)	687 (26.3%)
Positive repeat units (22862)	**14521** (63.5%)	10854 (47.5%)	5344 (23.4%)	2924 (12.8%)

Number of correctly detected proteins (sensitivity) and repeat units (accuracy) in the RepeatsDB-AFDB dataset for pLM-Repeat, HHrepID (MSA), HHrepID (Single), and RADAR at default threshold settings.
